# Retroviral Infections Affect Survival and Clutch Size of Female Wild Turkeys

**DOI:** 10.1002/ece3.73383

**Published:** 2026-04-08

**Authors:** Stephanie A. Shea, Matthew Gonnerman, Erik Blomberg, Kelsey Sullivan, Pauline L. Kamath

**Affiliations:** ^1^ Cooperative Extension University of Maine Orono Maine USA; ^2^ College of Agriculture and Natural Resources University of Maryland College Park Maryland USA; ^3^ Department of Wildlife Fisheries and Conservation Biology University of Maine Orono Maine USA; ^4^ Maine Department of Inland Fisheries and Wildlife Bangor Maine USA; ^5^ School of Food and Agriculture University of Maine Orono Maine USA

**Keywords:** coinfection, fitness, *Meleagris gallopavo*, reproduction, retrovirus, survival, wildlife disease

## Abstract

Pathogens can regulate or decimate free‐ranging wildlife populations. Wild turkeys ( *Meleagris gallopavo* ), which are widespread across the United States, southern Canada, and northern and central Mexico, are a prized upland gamebird that has experienced dramatic population growth and range expansion as the result of reintroduction campaigns. While increased abundance may promote disease transmission, little is known about the effects of pathogen infections on demographic metrics in wild turkeys. Lymphoproliferative disease virus (LPDV) and reticuloendotheliosis virus (REV) are oncogenic retroviruses that infect poultry and wild turkeys and can result in disease and mortality, though most infected individuals appear asymptomatic. We investigated whether retroviral infections influence wild turkey fitness by evaluating effects on female survival and several reproduction metrics. We live‐captured 163 female wild turkeys throughout central Maine, USA during three winters, from 2018 to 2020. We collected blood for LPDV and REV molecular diagnostics and attached a GPS or VHF transmitter to monitor survival and nesting. Infection with REV was associated with nearly half the cumulative annual survival probability, while LPDV‐infected hens laid an average of 1.4 fewer eggs per clutch. We detected no effects of retroviral infection on nest initiation, nesting propensity, or hatch rate, and coinfection was not associated with any measured demographic metric. These findings demonstrate that retroviral infections can negatively affect survival and clutch size in female wild turkeys even in the absence of overt disease, highlighting the importance of considering pathogen effects when evaluating the population dynamics of free‐ranging wildlife.

## Graphical abstract

We investigated the effects of two oncogenic retroviruses, lymphoproliferative disease virus (LPDV) and reticuloendotheliosis virus (REV), on survival and reproduction of wild turkeys in Maine. REV infection reduced female annual survival by nearly half, while LPDV‐infected hens laid fewer eggs, though other reproductive metrics were unaffected. These results show that retroviral infections can negatively impact wild turkey demographics even in the absence of overt disease symptoms.

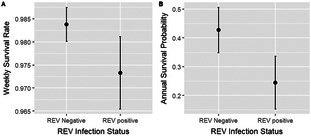

## 1 Introduction

Pathogens influence free‐ranging wildlife populations along a continuum of effects, ranging from little impact on individual fitness (Kilpatrick et al. 2006) to substantial population regulation (Dobson and Hudson 1992) or decline (McCallum et al. 2009; Dadam et al. 2019). More commonly, pathogens exert sublethal effects on host demographic metrics—such as survival and reproduction—without causing overt population crashes (Lachish et al. 2012; Palinauskas et al. 2018; Pigeault et al. 2018). Demographic consequences may arise through direct effects on survival (e.g., infection‐associated mortality; Palinauskas et al. 2018), reproduction (e.g., reduced number of chicks fledged; Pigeault et al. 2018), or both (Lachish et al. 2012). In other cases, hosts can tolerate pathogen infections with no apparent cost to survival or reproduction, as demonstrated in eastern bluebirds ( *Sialia sialis* ) following exposure to West Nile virus (Hill et al. 2010). These varied outcomes indicate that pathogen effects on wildlife are highly context‐dependent, shaped by the interplay of host traits, pathogen characteristics, and environmental factors.

Although pathogen‐driven population declines have received substantial attention, most wildlife studies report either sublethal or no detectable fitness effects of infection. This pattern reflects both true biological variability among host‐pathogen systems and a scarcity of studies that simultaneously assess the effects of multiple, coinfecting pathogen infections on demographic metrics, while accounting for individual host factors. Therefore, uncertainty remains about when pathogen infections warrant management intervention versus monitoring alone. Importantly, accurately predicting population‐level disease dynamics requires understanding how coinfections alter infection outcomes and fitness at the individual level, as interactions among parasites within hosts can scale up to influence transmission and population processes (Gorsich et al. 2018).

While pathogen prevalence models provide insights into host infection levels and spatiotemporal distributions, such datasets do not reveal the fitness consequences of infection at the individual‐ or population‐level, which are relevant for informing management strategies. For instance, while 90% of keelback snakes ( *Tropidonophis mairii* ) were infected with haemogregarine blood parasites, no negative effects on host fitness were observed, and management was unnecessary (Brown et al. 2006). Thus, to accurately describe the impacts of pathogens on population dynamics and predict the outcomes of disease management interventions, it is critical to employ a holistic approach that accounts for infection effects on fitness metrics, while also considering potentially confounding spatiotemporal and host factors. For example, simulation models that assessed demographic and epidemiology parameters in a managed versus unmanaged population of Tasmanian devils ( *Sarcophilus harrisii* ) revealed that selective culling would not be an effective strategy for reducing facial tumor disease, which is caused by an infectious cancer (Lachish et al. 2010).

Intrahost pathogen diversity, from coinfections, is another key but overlooked factor contributing to the dynamic nature of host‐pathogen relationships (Johnson and Hoverman 2012; Cassirer et al. 2018). Coinfection, which refers to more than one distinct infectious agent simultaneously infecting a single host (Cox 2001), can result in synergistic negative effects on fitness, such as increased host mortality (Johnson and Hoverman 2012). In addition, direct or indirect (i.e., through host immunity) within‐host interactions between multiple pathogen types can also lessen the effects of infection on the host (Knowles 2011), or the impact can appear to be neutral (i.e., no difference in mortality with single or multiple infections; Palinauskas et al. 2018). The complexity associated with multiple infections highlights the need to consider intrahost pathogen diversity and ecology when addressing infection outcomes (Telfer et al. 2010).

Lymphoproliferative disease virus (LPDV) and reticuloendotheliosis virus (REV) are avian oncogenic retroviral (family Retroviridae) pathogens that occur at the wildlife—domestic animal interface. Infection with LPDV was previously known to only infect and cause lymphoid tumors and mortality in domestic turkeys in Europe and Israel (Biggs et al. 1978; Biggs 1997). Reticuloendothleliosis virus was similarly first detected in a domestic turkey (Robinson and Twiehaus 1974) and is typically associated with runting syndrome or tumor growth and immunosuppression in poultry (Fadly et al. 2008). Furthermore, REV and avian leukosis virus (ALV) coinfection has a synergistic effect on chickens, increasing mortality, immunosuppression, and tumor growth (Dong et al. 2014). Lymphoproliferative disease virus is closely related to ALV (genus Alpharetrovius; Chajut et al. 1992), justifying concern for potential negative synergistic effects of LPDV‐REV coinfection similar to that seen in REV‐ALV coinfections (Dong et al. 2014).

The majority of concern and research on LPDV and REV has focused on domestic birds. However, Niedringhaus et al. (2019) recently issued a plea for further research to characterize the threat of LPDV and REV infection on wild turkey health following the detection of LPDV in wild turkeys ( *Meleagris gallopavo* ) in the United States (Allison et al. 2014). In addition to wild turkey health, there is concern for pathogen spillover to and from their domestic counterparts. Goodwin et al. (2024) recently verified that domestic turkeys could be experimentally infected with LPDV originating from wild turkeys. Furthermore, wild turkeys were the source of REV infection and caused mortality of nearly 50% of endangered Attwater's prairie chickens ( *Tympanuchus cupido attwateri* ) in a captive breeding facility (Stewart et al. 2019). While LPDV and REV coinfections in wild turkeys have been documented (Allison et al. 2014; MacDonald, Jardine, et al. 2019; MacDonald, Barta, et al. 2019; Shea et al. 2022; Cox et al. 2022; Adcock et al. 2024) and neoplasms have been observed in wild turkeys infected with LPDV or REV submitted as diagnostic cases (Allison et al. 2014; Niedringhaus et al. 2019; Adcock et al. 2024), it remains unknown how infections or coinfections of these pathogens affect wild turkey health and fitness. Furthermore, adult females, which incur the greatest energy costs associated with reproduction, including egg formation, incubation, and sole parental care (Porter 1977; Healy 1992), have a disproportionately higher likelihood of LPDV infection (Alger et al. 2017; Shea et al. 2022). Fitness metrics can vary in their relative contribution to population growth (i.e., their elasticity), which can vary by age class (Blomberg et al. 2021); thus, assessing whether age variation in infection prevalence translates to age‐dependent differences in pathogen‐related fitness consequences is warranted.

Declines in female survival may contribute to observed reductions in wild turkey populations (Lashley et al. 2025), yet the mechanisms driving this pattern are not well understood. Previous studies have evaluated the effects of predation (Reynolds and Swanson 2010; Niedzielski and Bowman 2015), legal harvest and illegal kills (Reynolds and Swanson 2010), vehicle collision (Kurzejeski et al. 1987), snow depth, food availability (Kane et al. 2007), season (Palmer et al. 1993; Niedzielski and Bowman 2015), land type (Spohr et al. 2004; Pollentier et al. 2014), behavior strategies during incubation (Lohr et al. 2020), dispersal distance, and home range size (Hubbard et al. 1999) on the survival of female wild turkeys, but these assessments rarely include pathogen infection (Palmer et al. 1993). Translocated individuals may be more susceptible to pathogen risk due to reduced genetic variation and naïve immune systems (Cunningham 1996). Additionally, harvesting combined with pathogen‐mediated fitness effects may reduce population productivity (Choisy and Rohani 2006). Considering how pathogen infections affect wild turkey survival is important given their status as a gamebird, and because infection may be enhanced and spread by reintroductions and subsequent population growth.

We evaluated the effects of REV, LPDV, and their coinfection on survival and reproduction of female wild turkeys. Although recent work has begun to examine short‐term reproductive and behavioral effects of LPDV infection (Goodman et al. 2025), additional research evaluating subclinical effects of LPDV on wild turkey fitness has been recommended (Goodwin et al. 2025). Our study addresses this need by evaluating longer‐term survival consequences of LPDV infection, assessing additional reproductive metrics, and providing the first assessment of REV and LPDV–REV coinfection effects on individual fitness. With a relatively high reported prevalence of both pathogens Maine wild turkeys (REV: 16%; LPDV: 59%) and a disproportionately higher probability of infection in adult females (Shea et al. 2022), our objectives were to (1) examine the effects of LPDV and REV infection and coinfection on demographic metrics in female wild turkeys and (2) determine whether these effects were age‐dependent. Specifically, the demographic metrics investigated include weekly survival rate, daily nest survival rate (DNSR), clutch size, nest initiation (Julian day of first egg laid), nesting propensity (rate at which a female nested if she was available to do so), and hatch rate (proportion of eggs available that hatched). These data provide valuable information to evaluate the risk of retroviral infections on wild turkey survival and reproduction, as well as the long‐term effects of these infections on population dynamics.

## 2 Materials and Methods

### 2.1 Field Methods

**FIGURE 1:**
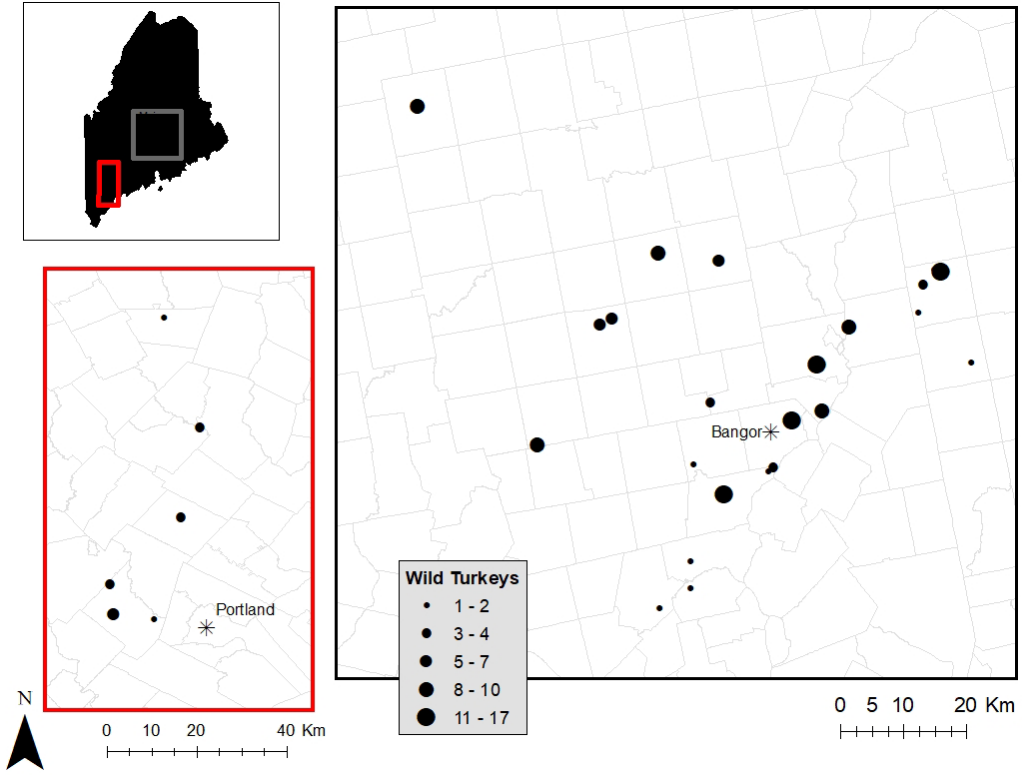
Capture site location of 163 wild turkeys fitted with GPS or VHF transmitters from 2018 to 2020 in Maine. Capture sites are sized by number captured.

We captured 163 live female turkeys over three winter seasons (Jan–Mar) using rocket or drop nets from 29 capture sites, located mostly in central and southern Maine (Figure 1). For all captured birds, we determined sex and recorded year of capture and body mass. We also determined age by observing primary wing feathers which are consistent in length with the middle feathers of the tail fan for adults but are not uniform in length compared to the middle feathers of the tail fan for subadults (< 1 year old; Dickson 1992). Each captured wild turkey was fitted with one of three unique transmitter models: (1) an 80 g VHF backpack‐style harness transmitter (*n* = 91; Advanced Telemetry Systems, Isanti, Minnesota), (2) a 90 g GPS backpack‐style harness transmitter with a built‐in VHF component (*n* = 46; Lotek Wireless Fish and Wildlife Monitoring, Newmarket, Ontario, CA), or (3) a 12 g VHF necklace transmitter (*n* = 26; Advanced Telemetry Systems, Isanti, Minnesota). For molecular diagnostics of LPDV and REV, whole blood was drawn from the brachial vein into an EDTA tube (1–5 mL; *n* = 129) or from a foot venipuncture into a heparin‐treated capillary tube and stored in queen's lysis buffer (~100 µL, *n* = 34). All capture, handling, and sampling of wild turkeys was approved by the University of Maine Institutional Animal Care and Use Committee (IACUC Protocol # A2017_11_03).

### 2.2 LPDV and REV Molecular Diagnostics

We used a molecular approach to determine the LPDV and REV proviral infection status of all sampled individuals. From the majority of blood samples (*n* = 127), we isolated the buffy coat layer by centrifuging for 15 min at 2500 RPM. In some cases (*n* = 36), when blood volume was too low for buffy coat optimization or when blood was collected via capillary tubes, we used whole blood. We extracted genomic DNA from both buffy coat and whole blood using Qiagen DNeasy Blood and Tissue Kits (Qiagen, Valencia, CA), following the manufacturer's instructions. For each extraction, we included a negative control and quantified DNA concentration using a NanoDrop One Spectrophotometer (Thermo Fisher Scientific, Wilmington, DE) or Qubit Fluorometer (Thermo Fisher Scientific, Waltham, MA). We determined retroviral infection status by PCR amplification of both a 413 base pair region of the LPDV *gag* gene and a 580 base pair region of the REV *pol* gene, following protocols described in Shea et al. (2022).

### 2.3 Monitoring Survival and Reproduction

We used both GPS and VHF transmitters to monitor survival of 163 individuals and 111 nests. We tracked all available individuals from their respective date of capture (first capture occurred on February 3rd, 2018) through the end of the study (November 14th, 2020). For VHF‐marked birds, we attempted to locate birds approximately once a week to record locations using a hand‐held three‐element directional antenna, where the live/dead status of each bird was determined based on the speed of the transmitter signal. We assessed survival of GPS‐marked individuals according to location information, with the date of mortality inferred according to sequential points at a single location. We recorded locations every hour during daylight (shifted periodically) from November through July, with an additional overnight location to record roosting sites. We downloaded data directly from transmitters regularly and uploaded information to Movebank (Kays et al. 2022). If either a GPS‐ or VHF‐tagged bird was suspected dead, the transmitter was approached to confirm mortality status. All birds were monitored with increased frequency for 2 weeks immediately following capture to assess potential for capture‐related mortality, and birds that died during this time were censored.

Female wild turkeys were monitored from April 15 to July 30 each year of the study for suspected nesting behavior. Particularly, we investigated the following metrics to evaluate the effects of pathogen infection on individual fitness: weekly survival rate, daily nest survival rate (DNSR), clutch size (number of eggs laid), nest initiation (Julian day of first egg laid), nesting propensity (rate at which a female nested if she was available to do so), and hatch rate (proportion of eggs available that hatched). Locations of VHF‐marked individuals were collected at least twice a week during the nesting period via short‐distance triangulation. If a hen was found alive in the same location during two successive visits, she was assumed to be on a nest. After 2 weeks, we approached the hen's location and flushed her to confirm nesting and locate the nest. We delayed flushing by this amount of time to decrease the chance of nest abandonment, which may be greatest when females are flushed during egg‐laying or early incubation (Götmark 1992). Nonetheless, nest abandonment occurred on eight occasions following flushing (further detailed in Gonnerman et al. 2022). We then floated 3–4 eggs to determine incubation stage, estimate the initiation date of the nest (Westerkov 1950), and predict a hatch date. Counting eggs during incubation reduced the likelihood of predation and underestimates. Furthermore, we continued to monitor the nest at least once a week, with a goal of 3 visits per week when possible. We increased visits around the suspected hatch date to better determine the actual hatch date. Once a hen was suspected to have left the nest, we approached the nest to assess its fate as hatched or failed.

Location data from GPS‐marked hens was downloaded weekly and point locations were reviewed in Google Earth. If we observed that a hen was making repeated visits to a single location around the same time of day, or had settled in a location she had previously visited regularly, we assumed she was nesting. Once the hen began regular movements or discontinued regular daily visits in the case of failure during the laying phase, we visited the suspected nest site to verify the nest and its fate.

### 2.4 Encounter History

We compiled weekly status (live/dead) for each GPS‐ and VHF‐marked wild turkey to develop a weekly encounter history for an individual, which included the week the turkey was captured, the last week it was found alive, the last week it was checked, and its final status at the end of the monitoring period. We increased monitoring frequency during the nesting season and similarly created an encounter history for DNSR. A subadult at capture remained a subadult through its first nesting season and was considered an adult starting August 1st of the year of capture to differentiate first‐time breeders.

### 2.5 Demographic Statistical Analyses

We evaluated the relationships between proviral infection status (REV, LPDV, coinfection), as independent variables, and several fitness metrics, including weekly survival rate, DNSR, clutch size for first and second nesting attempts, nest initiation for first and second nesting attempts, hatch rate, and nesting propensity for first and second nesting attempts. All analyses were conducted in RStudio (RStudio. 2021) using Program R (R Core Team 2021), and we used the *AICccmodavg* package (Mazerolle 2020) to employ a tiered AICc model selection approach. First, we considered nonpathogen factors that could explain variation in fitness metrics appropriate to each specific analysis; this included season, turkey age, and transmitter type when evaluating effects on survival, and turkey age, transmitter type, nest age, nest attempt, nest initiation, and/or nest year depending on the response variable associated with reproduction (DNSR, clutch size, nest initiation, hatch rate, or nesting propensity), when evaluating effects on reproduction. While we measured turkey body mass at time of capture, this was not included in analyses given that mass at capture is likely not relevant in subsequent years for survival or reproduction, and given our model structure. If there was support (< 2 delta AICc) for any models containing nonpathogen variables, the variables were included in a baseline model (only nonpathogen variables) and all other pathogen models. For the second AICc model selection (i.e., the pathogen model), we evaluated the baseline and pathogen models against an intercept‐only null model. For pathogen models, we included LPDV or REV infection status as a binary variable (0 = PCR negative, 1 = PCR positive), and a 4‐level coinfection categorical variable (represented as “coinf” in supplemental materials) with a level for each of the following: uninfected, infected with LPDV, infected with REV, infected with both LPDV and REV. We also included an age model when sample size allowed (with an age variable added to the baseline null model) because demographic estimates have been previously demonstrated to vary based on age in wild turkeys (Lehman et al. 2008; Pollentier et al. 2014). Lastly, age was also considered as a predictor in two independent models per pathogen variable, one that included an additive age term and one that included an age interaction term with each pathogen variable because our previous work revealed that adults are more likely to be infected than subadults (Shea et al. 2022), and age‐specific variation in pathogen effects can result in disproportional consequences in population growth.

Burnham and Anderson (2003) advised against using multiple notations of significance (e.g., reporting *p* values when using AICc model selection); therefore, we interpreted the significance of variables contained in supported models of the second AICc model selection by evaluating coefficients and their 95% confidence intervals (significance = confidence interval not overlapping zero). In linear models, AIC comparisons also account for an additional parameter estimating residual variance.

### 2.6 Evaluating Pathogen Effects on Weekly Survival Rate

We modeled weekly survival probability for females using the nest survival model in the *RMark* package (Laake 2013) in program R (R Core Team 2021). This known‐fate model type is commonly used for survival analyses for radio‐marked individuals and has been applied to turkeys in this manner previously (Collier et al. 2009), among other species. We chose this approach because it allowed for irregular monitoring of individuals, which best fit our study design by accommodating both staggered entry and exit of individuals continually throughout the study. Furthermore, we generally followed turkeys to death or end of study. An incomplete history likely represents a bird which either left the study area or whose transmitter stopped working. We flew planes with telemetry searching well beyond our study area to ensure it was not the former and truncated histories if we subsequently and reasonably assumed the latter. We exponentiated the weekly survival rate across 52 weeks to obtain an annual survival probability. For the first AICc model selection, we evaluated turkey age, season (winter = Janauary–March, spring = April–June, summer = July–September, and fall = October–December), and transmitter type (as either backpack‐harness style where we combined GPS and VHF models or necklace style) as predictors of survival, comparing these models against an intercept only null model. Since the model containing season better predicted survival than the null model, and Shea et al. (2022) also found evidence that LPDV infection varied seasonally, we hypothesized there might also be an interaction effect between season and infection status on survival. Therefore, for the second (pathogen) AICc model selection, in addition to comparing the age models specified above (interaction and additive term with each pathogen variable), we also included three additional season models, each with an interaction term between season and one of the pathogen variables (LPDV, REV, coinfection).

### 2.7 Evaluating Pathogen Effects on Reproduction

We modeled DNSR using the nest survival model in the *RMark* package (Laake 2013). The variables included in the initial nonpathogen AICc model selection were turkey age (at nesting), nest age (days), nest attempt (first or second), Julian day of nest initiation, and nest year. We also evaluated transmitter type since, when gathering nesting data, transmitter types (GPS vs. VHF) were expected to result in different levels of disturbance to hens. Lastly, we calculated overall nest success, defined as the percentage of initiated nests that survived to hatching.

We used AICc model selection to determine the most appropriate models for describing the impacts of pathogen infection on clutch size (number of eggs laid) and nest initiation (Julian day of first egg laid). Clutch size reportedly varies based on nest attempt (Roberts et al. 1995); thus, we conducted preliminary linear regression analyses and confirmed that nest attempt was a significant predictor of clutch size in our study. Nest initiation inherently varies based on nest attempt, as second nesting attempts chronologically follow first nest attempts. Therefore, we subset our data to analyze the first and second nest attempts separately for clutch size and nest initiation (third nest attempt was excluded from the analyses due to a sample size of one). The variables included in the initial nonpathogen AICc linear model selection for both clutch size and nest initiation were turkey age (at nesting) and year (of nesting). For clutch size, we also included two models containing either Julian day of nest initiation or the Julian day quadratic term. Clutch size (count data) was evaluated for residual normality. Residuals met the assumption of normality based on the Shapiro–Wilk test (*p* > 0.05) and inspection of Normal Q–Q Plots. For nest initiation of the second nesting attempt, the 4‐level coinfection categorical variable was not assessed in the second (pathogen) model selection due to small sample size. To further validate the clutch size results and assess the biological relevance, we used a simulation model to evaluate how recruitment would respond to a single loss in egg production by allowing clutch size to vary based on the middle 50% of our clutch sizes and allowing recruitment rate to vary (Table S1).

We examined the relationship between pathogen infection status and nesting propensity, defined as the rate at which a female nested if she was available to do so, for both first and second nesting attempts. We used AIC generalized linear model selection with a binomial distribution to determine if hen age or nest year affected nesting propensity (0 = did not nest, 1 = nested) for the initial nonpathogen model. To determine nesting propensity for first and second nests, we excluded individuals with VHF transmitters due to the higher potential for missed nests. We included any hen that was alive (available to nest) on the average Julian day of nest initiation specifically for each year and nesting attempt. For second nests, individuals were considered available to nest only if they had failed their previous attempt and were alive on the estimated average Julian day of nest initiation for that attempt in a given year. When analyzing nesting propensity for the first nesting attempt, the 4‐level coinfection categorical variable was not assessed in the second (pathogen) model selection due to small sample size. Similarly, only univariate pathogen models were assessed in the second (pathogen) model selection for the second nest attempt due to limited sample size.

Lastly, we assessed the effect of pathogen infection on hatch rate of successful nests for both VHF‐ and GPS‐marked birds. Hatch rate is defined as the ratio of the number of eggs that hatched out of the total number of eggs available to hatch (clutch size). We included hen age, nest year, nest initiation (two models containing either Julian day of nest initiation or the Julian day quadratic term), and nest attempt in the initial (nonpathogen) linear model selection. Due to small sample size, we only assessed univariate pathogen variables as an additive variable in the second model selection.

## 3 Results

### 3.1 Evaluating Pathogen Effects on Weekly Survival Rate

**FIGURE 2:**
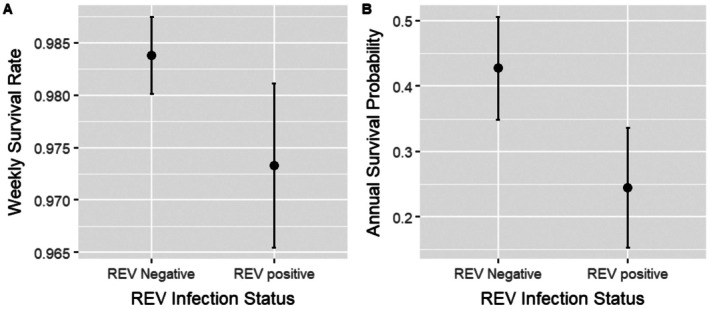
(A) Weekly survival rate and (B) cumulative annual survival probability based on REV infection status for 163 female wild turkeys captured and monitored over 3 years (2018–2020) in Maine. Estimates (with standard error bars) were derived from top performing models of weekly survival rate using AICc model selection.

We analyzed survival data of 163 female wild turkeys. Pathogen prevalence, including single and dual infections, is reported in Table 1. All infected individuals were outwardly asymptomatic, except for one LPDV/REV‐positive turkey with facial lesions that died 7 weeks postcapture. The top performing models, after the second model selection, included the additive terms of season, REV, and hen age (Tables S2 and S3). Thus, transmitter type was not a predictor of weekly survival rate, indicating transmitter placement (back or neck) or mass difference (~73 g) did not impact survival. Only REV infection was statistically significant (*β* = −0.510; 95% CI = −0.976 to −0.043). When averaged across season and hen age variables, an REV‐infected individual had a weekly survival rate of 0.973 (95% CI: 0.954–0.985), compared to a rate of 0.984 (95% CI: 0.975–0.989) for their uninfected counterparts (Figure 2A). This translates to an REV‐infected individual having nearly half (0.245; 95% CI = 0.0086–0.445) the cumulative annual survival probability of an uninfected individual (0.427; 95% CI = 0.268–0.574; Figure 2B).

**TABLE 1:** Pathogen prevalence in 163 wild turkey females that were included in the weekly survival rate assessment.

Pathogen	# Infected	Prevalence	95% CI
LPDV	117	71.8%	64.4%–78.1%
REV	38	23.3%	17.5%–30.4%
LPDV only	89	54.6%	46.9%–62.1%
REV only	10	6.1%	3.4%–10.9%
Both LPDV and REV	28	17.2%	12.2%–23.7%
Neither LPDV or REV	36	22.1%	16.4%–29.1%

### 3.2 Evaluating Pathogen Effects on Reproduction

**FIGURE 3:**
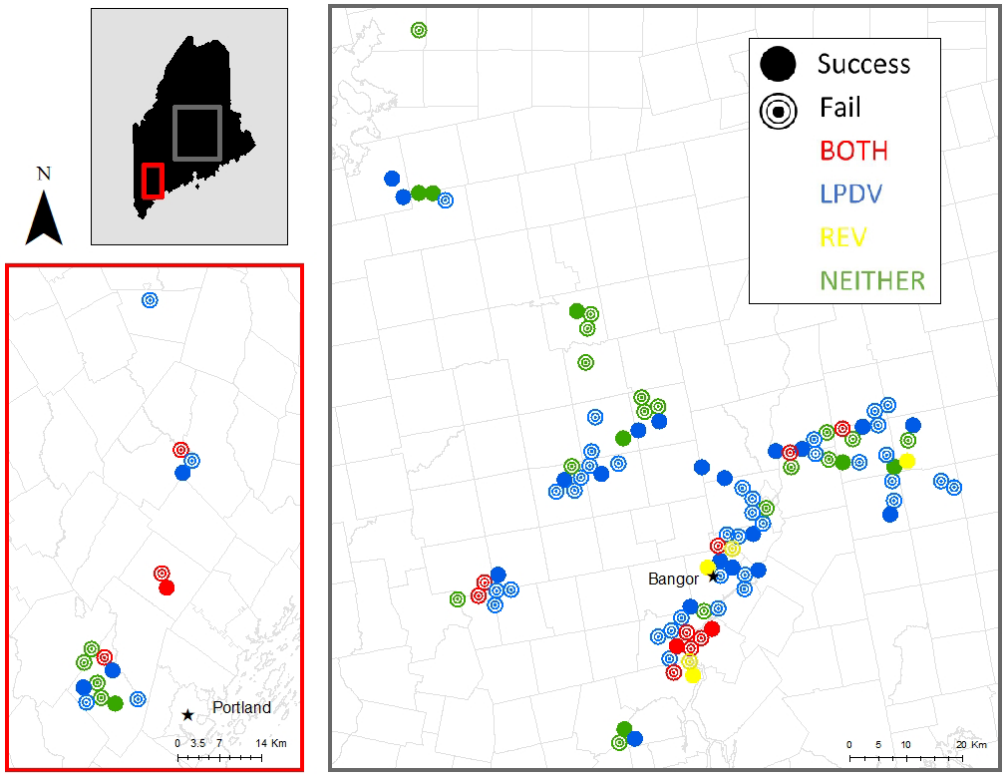
Nest locations for all nesting attempts across all 3 years (2018–2020) for female wild turkeys live‐captured in Maine. Nest sites are colored based on the infection status of the hen (LPDV only, REV only, both, or neither) and patterned according to nest fate (success or failure).

Overall nest success was 33.3% for 111 nests, with 30.2% of first nests (*n* = 96), 50% of second nests (*n* = 14), and the single third nest (100%; *n* = 1) being successful. Nest success was 33.8% and 32.4% for LPDV positive and negative hens, respectively, and 30.0% and 34.0% for REV positive and negative hens, respectively. Location and distribution of pathogen infection status and nest fate are shown in Figure 3. Pathogen prevalence and distribution of hens of 111 nests are reported in Table 2. The initial nonpathogen AICc model selection indicated nest age as a predictor of DNSR (Table S4), which was then included in all models during the following AICc model selection (Table S5). The model including transmitter type was not supported based on AICc, indicating that there was not a significant effect of nest disturbance (in the case of VHF‐tagged birds) on nest survival. For the second (pathogen) model selection, models containing REV, LPDV, turkey age, nest age, and the interaction of LPDV and turkey age were supported. We subsequently evaluated coefficients and confidence intervals of a model containing all supported variables, but only nest age was identified as a significant predictor of DNSR, where DNSR decreased with increasing nest age (*β* = −0.060; 95% CI = −0.085 to −0.035).

**TABLE 2:** Pathogen prevalence in 111 wild turkey hens of nests that were included in the daily nest survival rate assessment.

Pathogen	# Infected	Prevalence	95% CI
LPDV	77	69.4%	60.3%–77.2%
REV	20	18.0%	12.0%–26.2%
LPDV only	62	55.9%	46.6%–64.7%
REV Only	5	4.5%	1.9%–10.1%
Both LPDV and REV	15	13.5%	8.4%–21.1%
Neither LPDV or REV	29	26.1%	18.9%–35.0%

**FIGURE 4:**
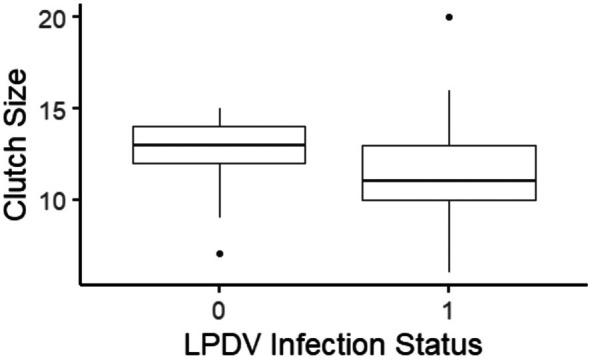
Boxplot displaying clutch size of 92 first nest attempts based on LPDV infection status (0: Uninfected, 1: Infected) over three nesting seasons (2018–2020). Estimates were derived from top performing models using AICc model selection.

We evaluated whether pathogen infection affected clutch size of 107 wild turkey nests (average clutch size by hen age and nesting attempt are shown in Table 3). For the first nest attempt, the variables contained in the top‐performing models of the initial nonpathogen model selection were nest initiation and nest initiation squared (Table S6). In addition to nest initiation variables, the top‐performing models in the pathogen model selection contained LPDV and turkey age as additive terms (Table S7). LPDV was a significant predictor of clutch size of first nest attempt (*β* = −1.43; 95% CI = −2.24 to −0.63), with LPDV‐infected hens laying an average of 1.43 (+/−0.41) fewer eggs than their uninfected counterparts (Figure 4). The simulation demonstrated that an average of one egg reduction in clutch size can result in an average of 7.5%–9% loss in recruitment. Turkey age and Julian day of nest initiation did not have a significant effect on clutch size. For the second nest attempt, age was contained in a top supported model in the first nonpathogen AICc model selection (Table S8), and models with pathogen variables were not supported in the second model selection (Table S9). While age was contained in a top‐performing model, the effect of age on clutch size was not significant, potentially due to the overall small sample size of second nests and uneven distribution of nests between subadults (*n* = 2) and adults (*n* = 12).

**TABLE 3:** Clutch size overall or by age or lymphoproliferative disease status for first, second, and third nest attempts with standard errors for 107 wild turkey nests over three nesting seasons (2018–2020) in Maine.

Cohort	Nest attempt	Mean clutch size	SE	Range	Sample size
All	All combined	11.5	0.2	6–20	107
	First	11.8	0.2	6–20	92
	Second	9.9	0.5	7–14	14
	Third	8	NA	NA	1
Adult	All combined	11.6	0.2	6–20	99
	First	11.8	0.2	6–20	86
	Second	10.2	0.5	7–14	12
	Third	8.0	NA	NA	1
Juvenile	All combined	10.0	1.1	7–15	8
	First	10.5	1.3	7–15	6
	Second	8.5	1.5	7–10	2
LPDV+	All combined	11.2	0.3	6–20	78
	First	11.1	0.3	6–20	69
	Second	9.6	0.5	7–12	8
	Third	8	NA	NA	1
LPDV−	All combined	12.3	0.4	7–15	29
	First	12.8	0.4	7–15	23
	Second	10.3	1.0	7–14	6

We investigated whether pathogen infection predicted nest initiation for 117 wild turkey nests (average nest initiation overall and by year, age, and attempt are in Table 4). For the first nest attempt, the model containing nest year performed better than the null model in the first nonpathogen AICc model selection (Table S10) and models including nest year, turkey age, LPDV, and REV were supported in the second (pathogen) model selection (Table S11). However, only nest year was found to be a significant predictor of nest initiation; nests in year 2019 were on average 8 days later than nests in 2018 (*β* = 8.19; 95% CI = 0.45–15.94). For the second nest attempt, only turkey age was included in the top‐performing model of the initial nonpathogen model selection (Table S12), and no additional models containing pathogen variables were supported in the second AICc model selection (Table S13). For the second nest attempt, subadults have an average nest initiation about 17 days earlier than adults (*β* = −17.07; 95% CI = −31.80 to −2.33), though this may be at least partly attributed to an uneven sample size of only 2 subadults compared to 15 adults. In conclusion, pathogen infections were not found to affect nest initiation in wild turkeys.

**TABLE 4:** Average nest initiation dates for 117 nests by year, overall, and by age for first, second, and third nest attempts of Wild Turkeys in Maine over 3 nesting seasons (2018–2020).

Cohort	Nest attempt	Nest initiation	SE	Sample size
2018	First	121.8	2.9	20
2019	First	129.3	2.0	32
2020	First	120.5	2.2	47
All	All combined	128.0	1.6	117
	First	123.6	1.4	99
	Second	151.1	2.6	17
	Third	174.0	NA	1
Adult	All combined	127.8	1.7	108
	First	123.2	1.5	92
	Second	153.1	2.3	15
	Third	174.0	NA	1
Juvenile	All combined	130.4	4.1	9
	First	128.9	4.8	7
	Second	136.0	9	2

We used data from 33 GPS‐backpacked hens to estimate nesting propensity and determine if there is variation based on retroviral infection status. Nesting propensity was estimated to be 0.80 (33/41) for first nests, 0.22 (5/23) for second nests, and 0.33 (1/3) for third nests. Two individuals from 2018 were available to nest in 2019 and 6 individuals from 2019 were available to nest in 2020; all other nests were from individuals captured during the winter immediately preceding the nesting season. For the first nest attempt, the model containing nest year performed better than the null model in the first nonpathogen AICc model selection (Table S14), and models including nest year and REV were supported in the second (pathogen) model selection (Table S15). However, neither variable was found to be a significant predictor of nesting propensity. For the second nesting attempt, the null model performed better than any other models in first and second model selection (Tables S16, S17). Thus, an effect of pathogen infection on nesting propensity was not detected.

There were 40 unhatched eggs out of a total of 419 eggs available to hatch (hatch rate of 85.8%) in 36 successful nests (2 subadults, 34 adults). In the initial model selection, only nest initiation was supported (Table S18) and we did not detect a pathogen effect on hatch rate in the second model selection (Table S19).

## 4 Discussion

Our study provides evidence to suggest retroviral (REV and LPDV) infections negatively affect female wild turkey fitness metrics. Infection with REV reduced annual survival by nearly half, while LPDV infection lowered clutch size, with infected hens laying an average of ~1.4 eggs fewer per clutch during the first nesting attempt. Asymptomatic pathogen infection was more common than outward disease; only one symptomatic individual was detected, which died within 7 weeks postcapture. Coinfection status did not seem to affect survival or reproduction. Overall, these results uncover the demographic consequences of asymptomatic retroviral infections and provide valuable data for parameterizing models that evaluate pathogen impacts on wild turkey population dynamics.

We identified a significant effect of REV infection on hen survival, with infected hens exhibiting approximately half the annual survival probability of uninfected hens (0.245 vs. 0.427). In contrast, we did not detect an effect of LPDV infection on survival, extending findings from the only previous study evaluating survival effects of LPDV, which reported no influence of infection on survival during incubation (Goodman et al. 2025). With almost a quarter of wild turkey hens infected with REV, the reduction in annual survival probability may translate to profound effects on population demography. Prevalence in our study is higher than previous surveys (nondiagnostic/clinical) that reported a prevalence of 8.5% (*n* = 331; Stewart et al. 2019), 2.9% (*n* = 70; Peterson et al. 2002), and 1.9% (*n* = 373; Cox et al. 2022) in Texas, USA, 11% in Kentucky (*n* = 36; Haynes et al. 2024), and 1.5% in Manitoba, Canada (MacDonald, Barta, et al. 2019). Infection with REV and subsequent clinical disease, including tumor growth and runting syndrome (Fadly et al. 2008), can cause direct mortality in chicken flocks, with mortality reaching up to 16% (Okoye et al. 1993). Reticuloendotheliosis virus also caused the death of nearly 50% of an Attwater's prairie chicken flock at a captive breeding facility (Stewart et al. 2019). In wild turkeys, REV has been associated with emaciation, poor nutritional condition, and neoplasms in the skin, liver, and spleen (Niedringhaus et al. 2019). The turkeys in this study were primarily outwardly asymptomatic at capture, which suggests that effects of REV may be subclinical, and may not cause direct mortality.

This effect of REV on survival may alternatively be indirect, via interaction with other known causes of host mortality. For instance, avifauna infected with malarial parasites incur an increased risk of predation compared with uninfected individuals (Møller and Nielsen 2007). Home range size has been associated with mortality risk for wild turkey hens (Hubbard et al. 1999), such that greater movements enable increased habitat sampling and refined selection that results in increased survival; thus, it is possible that REV infection limits hen habitat selection behaviors, thereby reducing home range size and increasing the subsequent mortality risk. Altered behavior has been demonstrated by house finches ( *Haemorhous mexicanus* ) infected with *Mycoplasma gallisepticum*, which were less mobile and more likely to be feeding alone than uninfected individuals (Dhondt et al. 2005; Hotchkiss et al. 2005). Infection may also increase the propensity to be hunted through effects on behavior or reactionary measures, which have been observed in other gamebirds (Jackson 1969). Furthermore, links have been found between pathogen infection and increased vehicle collisions in other host‐pathogen systems (Schwartz et al. 2020).

Reticuloendotheliosis virus is immunosuppressive in chickens (Fadly et al. 2008), facilitating subsequent infection by other pathogens that may not otherwise have been equipped to surpass host immune defenses, potentially affecting host survival. Specifically, REV infection has been shown to inhibit or reduce cytotoxic T cell proliferation, decreasing the host's ability to destroy tumor cells (Rup et al. 1979; Walker et al. 1983), though this suppression appears to be transient and may require continued viral replication (Rup et al. 1979). In addition, mortality, immunosuppression, and growth retardation caused by REV are exacerbated upon coinfection with another avian oncogenic retrovirus, ALV (Dong et al. 2014), a retrovirus closely related to LPDV (Chajut et al. 1992; Allison et al. 2014). While we did not see an impact of coinfection status on survival, there may be other pathogens not considered here that interact with REV in our wild turkey population. Because REV can be transmitted vertically (Witter and Purchase 1975), individuals may enter the population already infected, increasing the likelihood of early‐life coinfection and compounding downstream effects on host fitness and population dynamics.

We cannot discern whether the reduced survival rate of REV‐infected wild turkeys is representative of direct mortality, or if REV infection facilitates other stressors (such as environmental or additional parasites) that were ultimately the cause of mortality. While both direct and indirect factors are apparent in poultry, parsing the underlying mechanism of reduced survival is a common roadblock in research on wild populations (Burthe et al. 2008; Beldomenico et al. 2009). We hypothesized that a seasonal difference in survival according to pathogen status may speak to the seasonal pressures at a given point in time. For instance, hens on nests are more vulnerable; all turkeys in winter at the northern limit have limited resources due to snow, etc., which may increase their susceptibility to disease pressures. However, the interaction of REV and season was not supported, which may indicate the effects of REV infection are more uniform throughout the year. Little prior knowledge exists on the individual fitness effects of REV infection in free‐ranging avifauna, and we provide the first association with deleterious impacts on survival, though further attention is encouraged to discern underlying mechanisms.

Beyond impacts to morbidity and mortality, pathogens can influence host fitness by affecting reproductive success. Negative reproductive impacts may arise through reduced fecundity, compromised parental care, or vertical transmission of pathogens to offspring, leading to decreased offspring survival (Feore et al. 1997; Lachish et al. 2012; Markos and Abdela 2016). In our study, REV infection did not affect any measured reproductive demographic metric. Similarly, LPDV infection was not associated with most reproductive metrics we evaluated, consistent with the only previous study assessing reproductive effects of LPDV in wild turkeys, which reported no influence on nest success or hatch rate (Goodman et al. 2025). However, LPDV‐infected hens laid smaller clutches, a change that could translate to dampened fecundity. Fecundity‐related traits have been prioritized for improving population growth in wild turkeys, particularly because recruitment tends to be highly variable among populations (Roberts and Porter 1998; Pollentier et al. 2014).

The energetic demands of reproduction can make egg production vulnerable to reductions in hen condition associated with pathogen infection. We found that LPDV‐infected hens laid approximately 1.4 fewer eggs, translating to a ~12% reduction relative to the average clutch size of 11.8 eggs, which likely has a nontrivial effect on individual fitness. This pattern suggests that infection may impose energetic costs associated with immune response, potentially reducing resources available to support egg production or the laying period. Supporting this possibility, Goodman et al. (2025) found that LPDV‐infected hens traveled greater distances during the egg‐laying period than uninfected hens, which may reflect higher energetic expenditure or increased foraging effort to meet the demands of both reproduction and immune function.

Our conceptual simulation illustrated that even a reduction of one egg per clutch could reduce recruitment under a simplified scenario assuming a proportional relationship between clutch size and recruitment. For context, this effect size is comparable to the difference in mean clutch size between adult and juvenile females, suggesting that the reduction associated with LPDV infection may represent a biologically meaningful source of variation in production. Similarly, in another large‐bodied precocial bird, the greater snow goose (*Anser caerulescens atlanticus*), larger clutch sizes have been associated with both greater numbers and higher quality of fledglings (Lepage et al. 1998), highlighting the potential downstream influence of clutch size on reproductive outcomes. Experimental induction of the immune response in house sparrows ( *Passer domesticus* ) was linked with an increase in energy expenditure comparable to that required to produce a single egg (Martin et al. 2003). As a precocial species, wild turkeys incorporate a large amount of energy into the yolk (Carey et al. 1980) and require specific nutrients for egg development (Perrins 1996). Once developed, egg laying is a dangerous endeavor for birds; Perrins (1996) and Vangilder et al. (1987) found that only 80% of nests initiated reached incubation.

LPDV may be immunosuppressive in chickens (Payne 1998) and wild turkeys, based on pathogen coinfection rates (Shea et al. 2022). Therefore, it is plausible that tradeoffs with immunocompetence may reduce individual reproductive output (Svensson et al. 1998). Additionally, LPDV is an oncogenic retrovirus that can compromise hen condition. LPDV infection has been associated with emaciation and neoplasms in the liver, spleen, and skin (Niedringhaus et al. 2019). Although the effects of LPDV on reproductive metrics appear more modest than the effects of REV on survival, subclinical infection may still have meaningful consequences for individual reproductive performance.

The number of eggs laid is also positively correlated with the duration of the laying period (Williams et al. 1971) and, therefore, larger clutch sizes not only deplete nutrient sources, but may also increase predation risk during breeding. We found that nest age was also a significant predictor of DNSR, possibly reflecting either depletion of resources or increased vulnerability to predation as nests remain active for longer periods. In common eiders ( *Somateria mollissima* ), larger clutch size is correlated with reduced survival or breeding probability the following year (Descamps et al. 2009), suggesting a trade‐off in clutch size and future reproductive output and survival. Therefore, it is possible that wild turkeys burdened with LPDV must compensate with a reduction in clutch size to ensure survival and the prospect of future breeding (as an iteroparous species). Wild turkey population growth models are highly sensitive to poult survival (Pollentier et al. 2014), which we were not able to address in the current study. Further research considering poult survival would be critical for determining whether the effect of infection on clutch size translates to an ultimate reduction in fecundity, or if a diminished clutch size results in increased poult survival due to curtailed requirements of parental care as seen in great tits ( *Parus major* ; Lachish et al. (2012)).

Age‐structured differences in survival and reproduction play an important role in population dynamics (Gotelli 2008). Adults and females are more likely than subadults and males to be infected with LPDV (Alger et al. 2017; Shea et al. 2022), and seropositivity rates of REV also reportedly increased with age in chickens (Yang et al. 2017), highlighting age as a risk factor of infection. Therefore, we hypothesized that effects of LPDV and REV on individual demographic metrics may depend on age class, but we found no evidence for this. However, the higher prevalence of LPDV in adults may affect population‐level dynamics, for instance, due to the dampening effect of LPDV on reproductive output in the age cohort that contributes the most to reproduction (Roberts et al. 1995; Miller et al. 1998). A population growth analysis is warranted to determine the role of LPDV infection on long‐term population dynamics.

Coinfection can have varied effects on host fitness due to microbial infracommunity ecology and host immune response to multiple pathogens. We hypothesized that coinfection with LPDV and REV would intensify negative effects on demographic metrics through immune system suppression (Pedersen and Fenton 2007). Although we found approximately 13%–17% turkeys were coinfected, coinfection did not decrease survival or any reproductive metric relative to single pathogen or no infection. Retroviruses are chronically integrated into the host genome and, thus, are characterized by periods of both inactivity and active replication (Cloyd 1996; Justice IV and Beemon 2013; Rouzine et al. 2015), so it is possible that observable pathogen effects and interaction between coinfecting retroviruses are transient, whereby they do not share concurrent periods of activity. Alternatively, it is plausible that direct (resource) or apparent (immune‐mediated) competition (Cressler et al. 2016) is occurring that may reduce the active period of either virus, thereby reducing possible effects on immune function and fitness without altering the probability of proviral detection. It is also possible that additional microbes not considered in our study may be influencing infracommunity dynamics through within‐host processes such as competition or synergism (Johnson and Hoverman 2012). Expanding the number of pathogens surveyed and performing mechanistic experiments to understand within‐host microbial interactions could provide better context for deciphering coinfection dynamics.

Furthermore, we sampled wild turkeys in winter and assumed their LPDV and REV proviral DNA infection status remained constant across the entirety of the study, though this may not always reflect active infection (Cloyd 1996; Justice IV and Beemon 2013). It is likely that proviral infection status remained constant for those infected at capture due to viral insertion into the host genome; however, it may be less realistic for those uninfected at capture because they could become infected throughout the course of the study. In the case of the latter, the strength of the negative relationships between infection and fitness metrics observed in our study is likely conservative.

In conclusion, apparently asymptomatic retroviral infections in wild turkeys were associated with measurable individual fitness consequences that could ultimately influence population dynamics. Few studies on wild turkey survival and reproduction have incorporated host‐pathogen dynamics, yet our results demonstrate that infection status is a key component necessary for understanding population dynamics. Future research should prioritize integrating these findings into population growth models to determine potential population‐level impacts of retroviral infections, particularly by accounting for pathogen infection and the age‐specific importance of different fitness metrics. While female survival has been linked to wild turkey population growth (Lashley et al. 2025), harvest pressure is primarily directed toward males. Although we did not evaluate the effects of REV on male survival, similar reductions in survival among males could have implications for sustainable harvest management unless mitigated through adjustments to harvest regulations. Additionally, future work should consider the role of males in retroviral transmission, and subsequent infection‐mediated demographic effects on population dynamics. Furthermore, evaluating indicators of host immunocompetence and immune responses to LPDV and REV could provide insight into the mechanisms underlying infection‐associated reductions in individual fitness.

## Author Contributions

**Stephanie A. Shea:** conceptualization (equal), data curation (lead), formal analysis (lead), investigation (lead), writing – original draft (lead), writing – review and editing (lead). **Matthew Gonnerman:** data curation (equal), formal analysis (supporting), investigation (equal), writing – review and editing (supporting). **Erik Blomberg:** funding acquisition (equal), investigation (equal), supervision (supporting), writing – review and editing (supporting). **Kelsey Sullivan:** funding acquisition (equal), investigation (supporting), resources (lead), writing – review and editing (supporting). **Pauline L. Kamath:** conceptualization (equal), funding acquisition (equal), supervision (lead), writing – review and editing (lead).

## Funding

This project was funded by the Maine Department of Inland Fisheries and Wildlife, the National Wild Turkey Federation, the Maine State Chapter of the National Wild Turkey Federation, the Maine Outdoor Heritage Fund (Award No. 182‐01‐08), the University of Maine Graduate Student Government, and the USDA‐National Institute of Food and Agriculture Hatch Project Number ME0‐21908 and McIntire‐Stennis Project Number ME0‐41602 through the Maine Agricultural and Forest Experiment Station.

## Ethics Statement

All animal capture and handling procedures were approved by the University of Maine Institutional Animal Care and Use Committee (IACUC protocol # A2017_11_03) and conducted in accordance with relevant state and federal guidelines.

## Conflicts of Interest

The authors declare no conflicts of interest.

## Supporting information


**Table S1:** R code by line for post hoc analysis of biological impact of LPDV infection on clutch size.
**Table S2:** Initial AIC model selection comparing univariate models to identify relevant nonpathogen variables affecting weekly survival rate of female wild turkeys captured and monitored from 2018 to 2020 in Maine. Season was kept as a baseline null model in the second AIC model selection step.
**Table S3:** Second AIC model selection to identify pathogen variables affecting weekly survival rate of 163 female wild turkeys captured and monitored from 2018 to 2020 in Maine. All models except the intercept‐only model contain season as an explanatory variable, following Table S2. Variables determined to be significant upon interpretation of coefficients and their 95% confidence intervals in a model containing all supported variables (< 2 ΔAICc) are italicized.
**Table S4:** Initial AIC model selection comparing univariate models to identify relevant nonpathogen variables affecting daily nest survival rate of female wild turkeys captured and monitored from 2018 to 2020 in Maine. Nest age was kept as a baseline null model in the second AIC model selection step.
**Table S5:** Second AIC model selection models to identify pathogen variables affecting daily nest survival rate of female wild turkeys captured and monitored 2018–2020 in Maine. All models except the intercept‐only model contain nest age as an explanatory variable, following Table S4. Variables determined to be significant upon interpretation of coefficients and their 95% confidence intervals in a model containing all supported variables (< 2 ΔAICc) are italicized.
**Table S6:** Initial AIC model selection comparing models to identify nonpathogen variables affecting clutch size during the first nesting attempt of female wild turkeys captured and monitored from 2018 to 2020 in Maine. Nest initiation and nest initiation quadratic term were kept as a baseline model in the second AIC model selection step.
**Table S7:** Second AIC model selection models to identify pathogen variables affecting clutch size during the first nesting attempt of female wild turkeys captured and monitored from 2018 to 2020 in Maine. All models contain nest initiation and nest initiation quadratic term as explanatory variables, following Table S6 (for simplification, we only show the quadratic term). Variables determined to be significant upon interpretation of coefficients and their 95% confidence intervals in a model containing all supported variables (< 2 ΔAICc) are italicized.
**Table S8:** Initial AIC model selection comparing univariate models to identify relevant nonpathogen variables affecting clutch size during the second nesting attempt of female wild turkeys captured and monitored from 2018 to 2020 in Maine. Age was kept as a baseline null model in the second AIC model selection step.
**Table S9:** Second AIC model selection models to identify pathogen variables affecting clutch size during the second nesting attempt of female wild turkeys captured and monitored from 2018 to 2020 in Maine. All models contain turkey age as an explanatory variable, following Table S8. No variables were determined to be significant upon interpretation of coefficients and their 95% confidence intervals in a model containing all supported variables (< 2 ΔAICc).
**Table S10:** Initial AIC model selection comparing univariate models to identify relevant nonpathogen variables affecting nest initiation during the first nesting attempt of female wild turkeys captured and monitored from 2018 to 2020 in Maine. Nest year was kept as a baseline null model in the second AIC model selection step.
**Table S11:** Second AIC model selection models to identify pathogen variables affecting nest initiation during the first nesting attempt of female wild turkeys captured and monitored from 2018 to 2020 in Maine. All models contain nest year as explanatory variable, following Table S10. Variables determined to be significant upon interpretation of coefficients and their 95% confidence intervals in a model containing all supported variables (< 2 ΔAICc) are italicized.
**Table S12:** Initial AIC model selection comparing univariate models to identify relevant nonpathogen variables affecting nest initiation during the second nesting attempt of female wild turkeys captured and monitored from 2018 to 2020 in Maine. Age was kept as the baseline model in the second AIC model selection step.
**Table S13:** Second AIC model selection to identify pathogen variables affecting nest initiation during the second nesting attempt of female wild turkeys captured and monitored from 2018 to 2020 in Maine. All models contain age as an explanatory variable, following Table S12. Interaction between LPDV and age could not be assessed due to zero juveniles with LPDV and the 4‐category coinfection variable and age could not be assessed due to small sample size resulting in category singularities. Variables determined to be significant upon interpretation of coefficients and their 95% confidence intervals in a model containing all supported variables (< 2 ΔAICc) are italicized.
**Table S14:** Initial AIC model selection comparing univariate models to identify relevant nonpathogen variables affecting nesting propensity during the first nesting attempt of female wild turkeys captured and monitored from 2018 to 2020 in Maine. Nest year was kept as the baseline model in the second AIC model selection step.
**Table S15:** Second AIC model selection to identify pathogen variables affecting nesting propensity during the first nesting attempt of female wild turkeys captured and monitored from 2018 to 2020 in Maine. All models contain nest year as an explanatory variable, following Table S14. Interaction between the 4‐category coinfection variable and age could not be assessed due to small sample size resulting in category singularities. No variables were determined to be significant upon interpretation of coefficients and their 95% confidence intervals in a model. Containing all supported variables (< 2 ΔAICc).
**Table S16:** Initial AIC model selection comparing univariate models to identify relevant nonpathogen variables affecting nesting propensity during the second nesting attempt of female wild turkeys captured and monitored from 2018 to 2020 in Maine. The null model was kept as the baseline model in the second AIC model selection step.
**Table S17:** Second AIC model selection to identify pathogen variables affecting nesting propensity during the second nesting attempt of female wild turkeys captured and monitored from 2018 to 2020 in Maine. No variables were retained from the first model (Table S16). Due to small sample size, we only assessed univariate pathogen variables. Coincidentally, infection LPDV and REV mirrored each other where 18 were infected with LPDV and 5 were not, and 5 were infected with REV and 18 were not. No individual in this analysis was infected with REV alone (hence “Coinf” having 3 parameters). The null model performed the best, likely due to low sample size.
**Table S18:** Initial AIC model selection comparing univariate models to identify relevant nonpathogen variables affecting egg hatch rate of successful nests of female wild turkeys captured and monitored from 2018 to 2020 in Maine. Nest initiation and nest initiation quadratic term were kept as a baseline model in the second AIC model selection step.
**Table S19:** Second AIC model selection to identify pathogen variables affecting hatch rate of successful nests of female wild turkeys captured and monitored from 2018 to 2020 in Maine. All models contain nest initiation and nest initiation quadratic term as explanatory variables, following Table S18 (for simplification, we only show the quadratic term). Due to small sample size, we only assessed univariate pathogen variables as an additive variable with nest initiation. No variables were determined to be significant upon interpretation of coefficients and their 95% confidence intervals in a model containing all supported variables (< 2 ΔAICc).

## Data Availability

The data and R code are available in a Dryad repository accessible at the following link: https://doi.org/10.5061/dryad.5tb2rbpgm.

## References

[ece373383-bib-0001] Adcock, K. G. , R. D. Berghaus , C. C. Goodwin , et al. 2024. “Lymphoproliferative Disease Virus and Reticuloendotheliosis Virus Detection and Disease in Wild Turkeys ( *Meleagris gallopavo* ).” Journal of Wildlife Diseases 60: 139–150. 37972643 10.7589/JWD-D-23-00012

[ece373383-bib-0002] Alger, K. , E. Bunting , K. Schuler , and C. M. Whipps . 2017. “Risk Factors for and Spatial Distribution of Lymphoproliferative Disease Virus (LPDV) in Wild Turkeys ( *Meleagris gallopavo* ) in New York State, USA.” Journal of Wildlife Diseases 53: 499–508. 28328350 10.7589/2016-06-137

[ece373383-bib-0003] Allison, A. B. , M. Kevin Keel , J. E. Philips , et al. 2014. “Avian Oncogenesis Induced by Lymphoproliferative Disease Virus: A Neglected or Emerging Retroviral Pathogen?” Virology 450, no. 2014: 2–12. 24503062 10.1016/j.virol.2013.11.037 PMC3925403

[ece373383-bib-0004] Beldomenico, P. M. , S. Telfer , L. Lukomski , S. Gebert , M. Bennett , and M. Begon . 2009. “Host Condition and Individual Risk of Cowpox Virus Infection in Natural Animal Populations: Cause or Effect?” Epidemiology and Infection 137: 1295–1301. 19144246 10.1017/S0950268808001866 PMC2952828

[ece373383-bib-0005] Biggs, P. M. 1997. “Lymphoproliferative Disease of Turkeys, 10th Edition.” In Diseases of Poultry, edited by B. Calnek , H. Barnes , C. Beard , L. McDougald , and Y. Saif , 485–489. Iowa State University Press.

[ece373383-bib-0006] Biggs, P. M. , J. S. Mcdougall , J. A. Frazier , and B. S. Milne . 1978. “Lymphoproliferative Disease of Turkeys 1. Clinical Aspects.” Avian Pathology 7: 131–139. 18770365 10.1080/03079457808418265

[ece373383-bib-0007] Blomberg, E. J. , J. Tebbenkamp , S. Dunham , and D. Harrison . 2021. “Forest Management Legacies Affect Demographics and Population Dynamics of Spruce Grouse in Northern Maine.” Forest Ecology and Management 483: 118898.

[ece373383-bib-0008] Brown, G. P. , C. M. Shilton , and R. Shine . 2006. “Do Parasites Matter? Assessing the Fitness Consequences of Haemogregarine Infection in Snakes.” Canadian Journal of Zoology 84: 668–676.

[ece373383-bib-0009] Burnham, K. P. , and D. R. Anderson . 2003. Model Selection and Multimodel Inference: A Practical Information‐Theoretic Approach. 2nd ed, 496. Springer.

[ece373383-bib-0010] Burthe, S. , S. Telfer , M. Begon , M. Bennett , A. Smith , and X. Lambin . 2008. “Cowpox Virus Infection in Natural Field Vole *Microtus agrestis* Populations: Significant Negative Impacts on Survival.” Journal of Animal Ecology 77: 110–119. 18177331 10.1111/j.1365-2656.2007.01302.x PMC2970843

[ece373383-bib-0011] Carey, C. , H. Rahn , and P. Parisi . 1980. “Calories, Water, Lipid and Yolk in Avian Eggs.” Condor 82: 335–343.

[ece373383-bib-0012] Cassirer, E. F. , K. R. Manlove , E. S. Almberg , et al. 2018. “Pneumonia in Bighorn Sheep: Risk and Resilience.” Journal of Wildlife Management 82: 32–45.

[ece373383-bib-0013] Chajut, A. , R. Sarid , A. Yaniv , G. W. Smythers , S. R. Tronick , and A. Gazit . 1992. “The Lymphoproliferative Disease Virus of Turkeys Represents a Distinct Class of Avian Type‐C Retrovirus.” Gene 122: 349–354. 1283141 10.1016/0378-1119(92)90225-e

[ece373383-bib-0014] Choisy, M. , and P. Rohani . 2006. “Harvesting Can Increase Severity of Wildlife Disease Epidemics.” Proceedings of the Royal Society B 273: 2025–2034. 16846909 10.1098/rspb.2006.3554 PMC1635483

[ece373383-bib-0015] Cloyd, M. W. 1996. “Human Retroviruses.” In Medical Microbiology, edited by S. Baron , 4th ed., 1273. University of Texas Medical Branch at Galveston. 21413279

[ece373383-bib-0016] Collier, B. A. , K. B. Melton , J. B. Hardin , N. J. Silvy , and M. J. Peterson . 2009. “Impact of Reproductive Effort on Survival of Rio Grande Wild Turkey *Meleagris gallopavo intermedia* Hens in Texas.” Wildlife Biology 15: 370–379.

[ece373383-bib-0017] Cox, F. , J. Hardin , R. Dittmar , and D. Edwards . 2022. “Molecular Surveillance for Lymphoproliferative Disease Virus and Reticuloendotheliosis Virus in Rio Grande Wild Turkeys ( *Meleagris gallopavo intermedia* ) in Texas, USA. Wildlife Disease Association, Inc.” Journal of Wildlife Diseases 58: 909–913. 36305745 10.7589/JWD-D-22-00023

[ece373383-bib-0018] Cox, F. E. 2001. “Concomitant Infections, Parasites and Immune Responses.” Parasitol 122: S23–S38. 10.1017/s003118200001698x 11442193

[ece373383-bib-0019] Cressler, C. E. , D. V. McLeod , C. Rozins , J. Van Den Hoogen , and T. Day . 2016. “The Adaptive Evolution of Virulence: A Review of Theoretical Predictions and Empirical Tests.” Parasitol 143: 915–930. 10.1017/S003118201500092X PMC4873896 26302775

[ece373383-bib-0020] Cunningham, A. A. 1996. “Disease Risks of Wildlife Translocations.” Conservation Biology 10: 349–353.

[ece373383-bib-0021] Dadam, D. , R. A. Robinson , A. Clements , et al. 2019. “Avian Malaria‐Mediated Population Decline of a Widespread Iconic Bird Species.” Royal Society Open Science 6, no. 7: 182197. 31417708 10.1098/rsos.182197 PMC6689627

[ece373383-bib-0022] Descamps, S. , H. G. Gilchrist , J. Bêty , E. I. Buttler , and M. R. Forbes . 2009. “Costs of Reproduction in a Long‐Lived Bird: Large Clutch Size Is Associated With Low Survival in the Presence of a Highly Virulent Disease.” Biology Letters 5: 278–281. 19324661 10.1098/rsbl.2008.0704 PMC2665826

[ece373383-bib-0023] Dhondt, A. A. , S. Altizer , E. G. Cooch , et al. 2005. “Dynamics of a Novel Pathogen in an Avian Host: Mycoplasmal Conjunctivitis in House Finches.” Acta Tropica 94: 77–93. 15777638 10.1016/j.actatropica.2005.01.009

[ece373383-bib-0024] Dickson, J. G. 1992. The Wild Turkey Biology and Management, 480. Stackpole Books.

[ece373383-bib-0025] Dobson, A. P. , and P. J. Hudson . 1992. “Regulation and Stability of a Free‐Living Host‐Parasite System: Trichostrongylus Tenuis in Red Grouse. II. Population Models.” Journal of Animal Ecology 61: 487.

[ece373383-bib-0026] Dong, X. , S. Ju , P. Zhao , et al. 2014. “Synergetic Effects of Subgroup J Avian Leukosis Virus and Reticuloendotheliosis Virus Co‐Infection on Growth Retardation and Immunosuppression in SPF Chickens.” Veterinary Microbiology 172: 425–431. 25042879 10.1016/j.vetmic.2014.06.025

[ece373383-bib-0027] Fadly, A. M. , G. Zavala , and R. L. Witter . 2008. “Reticuloendotheliosis, 12th Edition.” In Diseases of Poultry, edited by Y. M. Saif , H. G. Barnes , J. R. Glisson , L. R. McDougald , L. K. Nolan , and D. E. Swayne , 568–588. Iowa State University Press.

[ece373383-bib-0028] Feore, S. M. , M. Bennett , J. Chantrey , T. Jones , D. Baxby , and M. Begon . 1997. “The Effect of Cowpox Virus Infection on Fecundity in Bank Voles and Wood Mice.” Proceedings of the Royal Society B: Biological Sciences 264: 1457–1461. 10.1098/rspb.1997.0202 PMC1688698 9364786

[ece373383-bib-0029] Gonnerman, M. , S. A. Shea , K. Sullivan , P. Kamath , and E. Blomberg . 2022. “Variation in Eastern Wild Turkey Nesting Phenology at Their Northern Range Limit.” Wildlife Society Bulletin 46, no. 2: e1278.

[ece373383-bib-0030] Goodman, P. E. , N. W. Bakner , D. L. Bakner , et al. 2025. “Influence of Lymphoproliferative Disease Virus on Behaviors of Female Eastern Wild Turkeys During Reproductive Periods.” Wildlife Society Bulletin 49, no. S1: e1632. 10.1002/wsb.1632.

[ece373383-bib-0031] Goodwin, C. C. , K. G. Adcock , A. B. Allison , M. G. Ruder , R. L. Poulson , and N. M. Nemeth . 2024. “Experimental Infection of Domestic Turkeys With Lymphoproliferative Disease Virus of North American Origin.” Veterinary Pathology 61: 562–573. 38415450 10.1177/03009858241231558

[ece373383-bib-0032] Goodwin, C. C. , A. B. Allison , M. G. Ruder , et al. 2025. “Current Understanding of Lymphoproliferative Disease Virus in Wild Turkeys.” Wildlife Society Bulletin 49, no. S1: e1644. 10.1002/wsb.1644.

[ece373383-bib-0033] Gorsich, E. E. , R. S. Etienne , J. Medlock , et al. 2018. “Opposite Outcomes of Coinfection at Individual and Population Scales.” Proceedings of the National Academy of SciencesUSA 115, no. 29: 745–7550. 10.1073/pnas.1801095115. PMC6055155 29967175

[ece373383-bib-0034] Gotelli, N. J. 2008. A Primer of Ecology. 4th ed, 291. Sinauer Associates, Inc.

[ece373383-bib-0035] Götmark, F. 1992. “The Effects of Investigator Disturbance on Nesting Birds.” In Current Ornothology, edited by D. M. Power , 63–104. Current Ornithology. Springer.

[ece373383-bib-0036] Haynes, E. , M. J. Yabsley , N. M. Nemeth , et al. 2024. “Health Assessment of Adult Male Eastern Wild Turkeys ( *Meleagris gallopavo silvestris* ) From Western Kentucky, USA.” Journal of Wildlife Diseases 60, no. 3: 660–669. 10.7589/JWD-D-23-00162. 38584308

[ece373383-bib-0037] Healy, W. M. 1992. “Behavior.” In The Wild Turkey: Biology and Management, edited by J. G. Dickson , 46–65. Stackpole Books.

[ece373383-bib-0038] Hill, G. E. , L. Siefferman , M. Liu , H. Hassan , and T. R. Unnasch . 2010. “The Effects of West Nile Virus on the Reproductive Success and Overwinter Survival of Eastern Bluebirds in Alabama.” Vector‐Borne and Zoonotic Diseases 10, no. 2: 159–163. 10.1089/vbz.2008.0211. 19589058 PMC2883462

[ece373383-bib-0039] Hotchkiss, E. R. , A. K. Davis , J. J. Cherry , and S. Altizer . 2005. “Mycoplasmal Conjunctivitis and the Behavior of Wild House Finches ( *Carpodacus mexicanus* ) at Bird Feeders.” Bird Behavior 17: 1–8.

[ece373383-bib-0040] Hubbard, M. W. , D. L. Garner , and E. E. Klaas . 1999. “Factors Influencing Wild Turkey Hen Survival in Southcentral Iowa.” Journal of Wildlife Management 63: 731–738.

[ece373383-bib-0041] Jackson, A. S. 1969. A Handbook for Bobwhite Quail Management in the West Texas Rolling Plains, 75. Texas Parks and Wildlife Department.

[ece373383-bib-0042] Johnson, P. T. J. , and J. T. Hoverman . 2012. “Parasite Diversity and Coinfection Determine Pathogen Infection Success and Host Fitness.” Proceedings of the National Academy of Sciences USA 109: 9006–9011. 10.1073/pnas.1201790109 PMC3384156 22615371

[ece373383-bib-0043] Justice, J., IV , and K. L. Beemon . 2013. “Avian Retroviral Replication.” Current Opinion in Virology 3: 664–669. 24011707 10.1016/j.coviro.2013.08.008 PMC3875226

[ece373383-bib-0044] Kane, D. F. , R. O. Kimmel , and W. E. Faber . 2007. “Winter Survival of Wild Turkey Females in Central Minnesota.” Journal of Wildlife Management 71: 1800–1807.

[ece373383-bib-0045] Kays, R. , S. C. Davidson , M. Berger , et al. 2022. “The Movebank System for Studying Global Animal Movement and Demography.” Methods in Ecology and Evolution 13: 419–431. 10.1111/2041-210X.13767.

[ece373383-bib-0046] Kilpatrick, A. M. , D. A. LaPointe , C. T. Atkinson , et al. 2006. “Effects of Chronic Avian Malaria (*Plasmodium relictum*) Infection on Reproductive Success of Hawaii Amakihi ( *Hemignathus virens* ).” Auk 123: 764–774.

[ece373383-bib-0047] Knowles, S. C. L. 2011. “The Effect of Helminth Co‐Infection on Malaria in Mice: A Meta‐Analysis.” International Journal for Parasitology 41: 1041–1051. 21777589 10.1016/j.ijpara.2011.05.009

[ece373383-bib-0048] Kurzejeski, E. W. , L. D. Vangilder , and J. B. Lewis . 1987. “Survival of Wild Turkey Hens in North Missouri.” Journal of Wildlife Management 51: 188–193.

[ece373383-bib-0049] Laake, J. L. 2013. RMark: An R Interface for Analysis of Capture‐Recapture Data With MARK. AFSC Processed Rep. 2013–01. Alaska Fisheries Science Center, NOAA, Seattle Washington USA.

[ece373383-bib-0050] Lachish, S. , M. B. Bonsall , B. Lawson , A. A. Cunningham , and B. C. Sheldon . 2012. “Individual and Population‐Level Impacts of an Emerging Poxvirus Disease in a Wild Population of Great Tits.” PLoS One 7, no. 11: e48545. 23185263 10.1371/journal.pone.0048545 PMC3504048

[ece373383-bib-0051] Lachish, S. , H. McCallum , D. Mann , C. E. Pukk , and M. E. Jones . 2010. “Evaluation of Selective Culling of Infected Individuals to Control Tasmanian Devil Facial Tumor Disease.” Conservation Biology 24: 841–851. 20088958 10.1111/j.1523-1739.2009.01429.x

[ece373383-bib-0052] Lashley, M. A. , M. C. Chitwood , A. K. Moeller , et al. 2025. “Decreased Female Survival May Help Explain Wild Turkey Population Decline.” Wildlife Society Bulletin 49, no. S1: e1642. 10.1002/wsb.1642.

[ece373383-bib-0053] Lehman, C. P. , M. A. Rumble , L. D. Flake , and D. J. Thompson . 2008. “Merriam's Turkey Nest Survival and Factors Affecting Nest Predation by Mammals.” Journal of Wildlife Management 72: 1765–1774.

[ece373383-bib-0054] Lepage, D. , G. Gauthier , and A. Desrochers . 1998. “Larger Clutch Size Increases Fledging Success and Offspring Quality in a Precocial Species.” Journal of Animal Ecology 67: 210–216.

[ece373383-bib-0055] Lohr, A. K. , J. A. Martin , G. T. Wann , B. S. Cohen , B. A. Collier , and M. J. Chamberlain . 2020. “Behavioral Strategies During Incubation Influence Nest and Female Survival of Wild Turkeys.” Ecology and Evolution 10: 11752–11765. 10.1002/ece3.6812. 33144998 PMC7593161

[ece373383-bib-0056] MacDonald, A. M. , J. R. Barta , M. McKay , et al. 2019. “Lymphoproliferative Disease Virus in Wild Turkeys ( *Meleagris gallopavo* ) From Manitoba and Quebec, Canada.” Avian Diseases 63: 506–510. 31967435 10.1637/aviandiseases-D-19-00102

[ece373383-bib-0057] MacDonald, A. M. , C. M. Jardine , J. Bowman , L. Susta , and N. M. Nemeth . 2019. “Detection of Lymphoproliferative Disease Virus in Canada in a Survey for Viruses in Ontario Wild Turkeys ( *Meleagris gallopavo* ).” Journal of Wildlife Diseases 55: 113–122. 30124393 10.7589/2018-01-013

[ece373383-bib-0058] Markos, T. , and N. Abdela . 2016. “Epidemiology and Economic Importance of Pullorum Disease in Poultry: A Review.” Global Veterinaria 17: 228–237.

[ece373383-bib-0059] Martin, L. B. , A. Scheuerlein , and M. Wikelski . 2003. “Immune Activity Elevates Energy Expenditure of House Sparrows: A Link Between Direct and Indirect Costs?” Proceedings of the Royal Society of London. Series B: Biological Sciences 270: 153–158. 10.1098/rspb.2002.2185 PMC1691219 12590753

[ece373383-bib-0060] Mazerolle, M. 2020. “AICcmodavg: Model Selection and Multimodel Inference Based on (Q)AIC(c).” https://cran.r‐project.org/web/packages/AICcmodavg/AICcmodavg.pdf.

[ece373383-bib-0061] McCallum, H. , M. Jones , C. Hawkins , et al. 2009. “Transmission Dynamics of Tasmanian Devil Facial Tumor Disease May Lead to Disease‐Induced Extinction.” Ecology 90: 3379–3392. 20120807 10.1890/08-1763.1

[ece373383-bib-0062] Miller, D. A. , B. D. Leopold , and G. A. Hurst . 1998. “Reproductive Characteristics of a Wild Turkey Population in Central Mississippi.” Journal of Wildlife Management 62: 903–910.

[ece373383-bib-0063] Møller, A. P. , and J. T. Nielsen . 2007. “Malaria and Risk of Predation: A Comparative Study of Birds.” Ecology 88: 871–881. 17536704 10.1890/06-0747

[ece373383-bib-0064] Niedringhaus, K. D. , N. M. Nemeth , H. S. Sellers , J. D. Brown , and H. M. A. Fenton . 2019. “Multicentric Round Cell Neoplasms and Their Viral Associations in Wild Turkeys ( *Meleagris gallopavo* ) in the Southeastern United States.” Veterinary Pathology 56: 915–920. 31345138 10.1177/0300985819864306

[ece373383-bib-0065] Niedzielski, B. , and J. Bowman . 2015. “Survival and Cause‐Specific Mortality of the Female Eastern Wild Turkey at Its Northern Range Edge.” Wildlife Research 41: 545–551. 10.1071/WR14061.

[ece373383-bib-0066] Okoye, J. O. A. , W. Ezema , and J. N. Agoha . 1993. “Naturally Occurring Clinical Reticuloendotheliosis in Turkeys and Chickens.” Avian Pathology 22: 237–244. 18671014 10.1080/03079459308418917

[ece373383-bib-0067] Palinauskas, V. , R. Žiegytė , J. Šengaut , and R. Bernotienė . 2018. “Different Paths – The Same Virulence: Experimental Study on Avian Single and Co‐Infections With Plasmodium Relictum and Plasmodium Elongatum.” International Journal for Parasitology 48: 1089–1096. 30367860 10.1016/j.ijpara.2018.08.003

[ece373383-bib-0068] Palmer, W. E. , G. A. Hurst , J. E. Stys , D. R. Smith , and J. D. Burk . 1993. “Survival Rates of Wild Turkey Hens in Loblolly Pine Plantations in Mississippi.” Journal of Wildlife Management 57: 783–789.

[ece373383-bib-0069] Payne, L. N. 1998. “Retrovirus‐Induced Disease in Poultry.” Poultry Science 77: 1204–1212. 10.1093/ps/77.8.1204 9706091

[ece373383-bib-0070] Pedersen, A. B. , and A. Fenton . 2007. “Emphasizing the Ecology in Parasite Community Ecology.” Trends in Ecology & Evolution 22: 133–139. 17137676 10.1016/j.tree.2006.11.005

[ece373383-bib-0071] Perrins, C. M. 1996. “Eggs, Egg Formation and the Timing of Breeding.” Ibis 138: 2–15.

[ece373383-bib-0072] Peterson, M. J. , R. Aguirre , P. J. Ferro , et al. 2002. “Infectious Disease Survey of Rio Grande Wild Turkeys in the Edwards Plateau of Texas.” Journal of Wildlife Diseases 38, no. 4: 826–833. 10.7589/0090-3558-38.4.826. 12528453

[ece373383-bib-0073] Pigeault, R. , C.‐S. Cozzarolo , R. Choquet , et al. 2018. “Haemosporidian Infection and Co‐Infection Affect Host Survival and Reproduction in Wild Populations of Great Tits.” International Journal for Parasitology 48: 1079–1087. 30391229 10.1016/j.ijpara.2018.06.007

[ece373383-bib-0074] Pollentier, C. D. , S. D. Hull , and R. S. Lutz . 2014. “Eastern Wild Turkey Demography: Sensitivity of Vital Rates Between Landscapes.” Journal of Wildlife Management 78: 1372–1382.

[ece373383-bib-0075] Porter, W. F. 1977. “Reproductive Success, Body Condition, and Survival of Female Wild Turkeys.” Journal of Wildlife Management 41, no. 1: 53–63.

[ece373383-bib-0076] R Core Team . 2021. R: A Language and Environment for Statistical Computing. R Foundation for Statistical computing.

[ece373383-bib-0077] Reynolds, M. C. , and D. A. Swanson . 2010. “Survival of Female Wild Turkeys in Southeastern Ohio.” Wildlife Society Bulletin 2010: 149–155. 10.1002/j.2328-5540.2010.tb00350.x.

[ece373383-bib-0078] Roberts, S. D. , J. M. Coffey , and W. F. Porter . 1995. “Survival and Reproduction of Female Wild Turkeys in New York.” Journal of Wildlife Management 59: 437–447.

[ece373383-bib-0079] Roberts, S. D. , and W. F. Porter . 1998. “Relation Between Weather and Survival of Wild Turkey Nests.” Journal of Wildlife Management 62: 1492–1498.

[ece373383-bib-0080] Robinson, F. R. , and M. J. Twiehaus . 1974. “Historical Note: Isolation of the Avian Reticuloendothelial Virus (Strain T).” Avian Diseases 18: 278–288. 4828587

[ece373383-bib-0081] Rouzine, I. M. , A. D. Weinberger , and L. S. Weinberger . 2015. “An Evolutionary Role for HIV Latency in Enhancing Viral Transmission.” Cell 160: 1002–1012. 25723173 10.1016/j.cell.2015.02.017 PMC4488136

[ece373383-bib-0082] RStudio . 2021. RStudio: Integrated Development for R. RStudio, PBC.

[ece373383-bib-0083] Rup, B. J. , J. L. Spence , J. D. Hoelzer , et al. 1979. “Immunosuppression Induced by Avian Reticuloendotheliosis Virus: Mechanism of the Suppressor Cell.” Journal of Immunology 123: 1362–1370. 224111

[ece373383-bib-0084] Schwartz, A. L. W. , F. M. Shilling , and S. E. Perkins . 2020. “The Value of Monitoring Wildlife Roadkill. European Journal of Wildlife Research.” European Journal of Wildlife Research 66, no. 1: 18.

[ece373383-bib-0085] Shea, S. A. , M. Gonnerman , E. Blomberg , K. Sullivan , P. Milligan , and P. L. Kamath . 2022. “Pathogen Survey and Predictors of Lymphoproliferative Disease Virus Infection in Wild Turkeys ( *Meleagris gallopavo* ).” Journal of Wildlife Diseases 58: 537–549. 35704504 10.7589/JWD-D-21-00152

[ece373383-bib-0086] Spohr, S. M. , F. A. Servello , D. J. Harrison , and D. W. May . 2004. “Survival and Reproduction of Female Wild Turkeys in a Suburban Environment.” Northeastern Naturalist 11, no. 4: 363–374.

[ece373383-bib-0087] Stewart, B. , C. Trautman , F. Cox , et al. 2019. “Survey of Reticuloendotheliosis Virus in Wild Turkeys ( *Meleagris gallopavo* ) in Texas, USA.” Journal of Wildlife Diseases 55, no. 3: 689–693. 10.7589/2018-08-187. 30557122

[ece373383-bib-0088] Svensson, E. , L. Råberg , C. Koch , and D. Hasselquist . 1998. “Energetic Stress, Immunosuppression and the Costs of an Antibody Response.” Functional Ecology 12: 912–919.

[ece373383-bib-0089] Telfer, S. , X. Lambin , R. Birtles , et al. 2010. “Species Interactions in a Parasite Community Drive Infection Risk in a Wildlife Population.” Science 330: 243–246. 20929776 10.1126/science.1190333 PMC3033556

[ece373383-bib-0090] Vangilder, L. D. , E. W. Kurzejeski , V. L. Kimmel‐Truitt , and J. B. Lewis . 1987. “Reproductive Parameters of Wild Turkey Hens in North Missouri.” Journal of Wildlife Management 51: 535.

[ece373383-bib-0091] Walker, M. H. , B. J. Rup , A. S. Rubin , and H. R. Bose . 1983. “Specificity in the Immunosuppression Induced by Avian Reticuloendotheliosis Virus.” Infection and Immunity 40: 225–235. 6187691 10.1128/iai.40.1.225-235.1983 PMC264840

[ece373383-bib-0092] Westerkov, K. 1950. “Methods for Determining the Age of Game Bird Eggs.” Journal of Wildlife Management 14, no. 1: 56–67.

[ece373383-bib-0093] Williams, L. E. , D. H. Austin , T. E. Peoples , and R. W. Phillips . 1971. “Laying Data and Nesting Behavior of Wild Turkeys.” Proceedings of the Annual Conference, Southeastern Association of Fish and Wildlife Agencies 25: 90–106.

[ece373383-bib-0094] Witter, R. L. , and H. G. Purchase . 1975. “Studies on the Vertical Transmission of Reticuloendotheliosis Virus.” Avian Diseases 19, no. 4: 716–724. 2546528

[ece373383-bib-0095] Yang, Y. , J. Zhao , Z. Ma , M. Xu , J. Xue , and G. Zhang . 2017. “Serological Survey of Reticuloendotheliosis Virus Infection in Chickens in China in 2005 to 2015.” Poultry Science 96: 3893–3895. 10.3382/ps/pex209 29050414

