# Supplementary material for: Retroviral Infections Affect Survival and Clutch Size of Female Wild Turkeys
Source: Ecol Evol. 2026 Apr 8;16(4):e73383. doi: 10.1002/ece3.73383 (PMC13058719; doi:10.1002/ece3.73383)
Supplement: Supplementary file 1 — Table S1: R code by line for post hoc analysis of biological impact of LPDV infection on clutch size. Table S2: Initial AIC model selection comparing univariate models to identify relevant nonpathogen variables affecting weekly survival rate of female wild turkeys captured and monitored from 2018 to 2020 in Maine. Season was kept as a baseline null model in the second AIC model selection step. Table S3: Second AIC model selection to identify pathogen variables affecting weekly survival rate of 163 female wild turkeys captured and monitored from 2018 to 2020 in Maine. All models except the intercept‐only model contain season as an explanatory variable, following Table S2. Variables determined to be significant upon interpretation of coefficients and their 95% confidence intervals in a model containing all supported variables (< 2 ΔAICc) are italicized. Table S4: Initial AIC model selection comparing univariate models to identify relevant nonpathogen variables affecting daily nest survival rate of female wild turkeys captured and monitored from 2018 to 2020 in Maine. Nest age was kept as a baseline null model in the second AIC model selection step. Table S5: Second AIC model selection models to identify pathogen variables affecting daily nest survival rate of female wild turkeys captured and monitored 2018–2020 in Maine. All models except the intercept‐only model contain nest age as an explanatory variable, following Table S4. Variables determined to be significant upon interpretation of coefficients and their 95% confidence intervals in a model containing all supported variables (< 2 ΔAICc) are italicized. Table S6: Initial AIC model selection comparing models to identify nonpathogen variables affecting clutch size during the first nesting attempt of female wild turkeys captured and monitored from 2018 to 2020 in Maine. Nest initiation and nest initiation quadratic term were kept as a baseline model in the second AIC model selection step. Table S7: Second AIC model s [file ECE3-16-e73383-s001.docx]

Table S1. R code by line for post hoc analysis of biological impact of LPDV infection on clutch size.

| Step | Code |
| --- | --- |
| 1 | Require(dplyr) |
| 2 | mean.egg <- 10:13 |
| 3 | mean.reduct <- 1 |
| 4 | brood.surv <- seq(.1,.5, .1) |
| 5 | expand.grid(Eggs = mean.egg,  EggReduction = mean.reduct,  Recruitment = brood.surv) %>% |
| 6 | rowwise() %>% |
| 7 | mutate(TotRecruit = mean(rbinom(10000, Eggs, Recruitment)),  ReductRecruit = mean(rbinom(10000,Eggs-EggReduction,Recruitment))) %>% |
| 8 | mutate(Loss = TotRecruit - ReductRecruit,  PercentLoss = 1-ReductRecruit/TotRecruit) %>% |
| 9 | PercentLoss = 1-ReductRecruit/TotRecruit) %>% |
| 10 | View() |

Table S2. Initial AIC model selection comparing univariate models to identify relevant non-pathogen variables affecting weekly survival rate of female wild turkeys captured and monitored from 2018–2020 in Maine. Season was kept as a baseline null model in the second AIC model selection step.

| **Model** | ***k*** | **AICc** | **ΔAICc** | **Weight** | **Dev.** |
| --- | --- | --- | --- | --- | --- |
| Season | 4 | 930.940 | 0.000 | 0.778 | 922.933 |
| Null | 1 | 934.614 | 3.674 | 0.124 | 932.613 |
| Age | 2 | 936.396 | 5.455 | 0.051 | 932.393 |
| Trans.Type | 2 | 936.555 | 5.615 | 0.047 | 932.553 |

| **Model** | ***k*** | **AICc** | **ΔAICc** | **Weight** | **Dev.** |
| --- | --- | --- | --- | --- | --- |
| Season + *REV* | 5 | 928.618 | 0.000 | 0.324 | 918.607 |
| Season + Age + REV | 6 | 930.215 | 1.598 | 0.146 | 918.200 |
| Season | 4 | 930.940 | 2.322 | 0.101 | 922.933 |
| Season + Coinf | 7 | 931.260 | 2.642 | 0.086 | 917.239 |
| Season + LPDV | 5 | 931.429 | 2.811 | 0.079 | 921.418 |
| Season + Age + REV + REV*Age | 7 | 931.797 | 3.179 | 0.066 | 917.776 |
| Season + Age | 5 | 932.413 | 3.795 | 0.049 | 922.402 |
| Season + Age + Coinf | 8 | 933.149 | 4.531 | 0.034 | 917.123 |
| Season + Age + LPDV | 6 | 933.270 | 4.652 | 0.032 | 921.254 |
| Season + REV + REV*Season | 8 | 933.563 | 4.945 | 0.027 | 917.537 |
| Season + Age + LPDV + LPDV*Adult | 7 | 934.087 | 5.469 | 0.021 | 920.067 |
| Null | 1 | 934.614 | 5.996 | 0.016 | 932.613 |
| Season + LPDV + LPDV*Season | 8 | 935.393 | 6.775 | 0.011 | 919.366 |
| Season + Age + Coinf + Coinf*Age | 11 | 936.434 | 7.816 | 0.006 | 914.386 |
| Season + Coinf + Season * Coinf | 16 | 938.959 | 10.341 | 0.002 | 906.858 |

Table S3. Second AIC model selection to identify pathogen variables affecting weekly survival rate of 163 female wild turkeys captured and monitored from 2018–2020 in Maine. All models except the intercept-only model contain season as an explanatory variable, following Table S2. Variables determined to be significant upon interpretation of coefficients and their 95% confidence intervals in a model containing all supported variables (<2 ΔAICc) are italicized.

Table S4. Initial AIC model selection comparing univariate models to identify relevant non-pathogen variables affecting daily nest survival rate of female wild turkeys captured and monitored from 2018–2020 in Maine. Nest age was kept as a baseline null model in the second AIC model selection step.

| **Model** | ***k*** | **AICc** | **ΔAICc** | **Weight** | **Dev.** |
| --- | --- | --- | --- | --- | --- |
| Nest.Age | 2 | 535.244 | 0.000 | 1.000 | 531.238 |
| Null | 1 | 553.170 | 17.926 | 0.000 | 551.168 |
| Age | 2 | 554.053 | 18.809 | 0.000 | 550.048 |
| Nest.Attempt | 3 | 554.113 | 18.870 | 0.000 | 548.103 |
| Nest.Initiation | 2 | 555.035 | 19.791 | 0.000 | 551.030 |
| Trans.Type | 3 | 556.775 | 21.531 | 0.000 | 550.764 |
| Nest.Year | 3 | 557.157 | 21.913 | 0.000 | 551.147 |

Table S5. Second AIC model selection models to identify pathogen variables affecting daily nest survival rate of female wild turkeys captured and monitored 2018–2020 in Maine. All models except the intercept-only model contain nest age as an explanatory variable, following Table S4. Variables determined to be significant upon interpretation of coefficients and their 95% confidence intervals in a model containing all supported variables (<2 ΔAICc) are italicized

| **Model** | ***k*** | **AICc** | **ΔAICc** | **Weight** | **Dev.** |
| --- | --- | --- | --- | --- | --- |
| *Nest.Age* + Age | 3 | 534.828 | 0.000 | 0.204 | 528.817 |
| Nest.Age | 2 | 535.244 | 0.416 | 0.166 | 531.238 |
| Nest.Age + Age + LPDV + LPDV * Age | 5 | 535.271 | 0.443 | 0.164 | 525.244 |
| Nest.Age + Age + REV | 4 | 536.480 | 1.652 | 0.089 | 528.462 |
| Nest.Age + Age + LPDV | 4 | 536.777 | 1.949 | 0.077 | 528.759 |
| Nest.Age + REV | 3 | 536.830 | 2.002 | 0.075 | 530.820 |
| Nest.Age + LPDV | 3 | 537.124 | 2.296 | 0.065 | 531.113 |
| Nest.Age + Age + Coinf | 6 | 537.653 | 2.826 | 0.050 | 525.616 |
| Nest.Age + Age + REV + REV*Age | 5 | 537.877 | 3.050 | 0.044 | 527.851 |
| Nest.Age + Coinfection | 5 | 537.923 | 3.095 | 0.043 | 527.896 |
| Nest.Age + Age + Coinf + Coinf* Age | 9 | 539.241 | 4.413 | 0.022 | 521.162 |
| Null | 1 | 553.170 | 18.342 | 0.000 | 551.168 |

Table S6. Initial AIC model selection comparing models to identify non-pathogen variables affecting clutch size during the first nesting attempt of female wild turkeys captured and monitored from 2018–2020 in Maine. Nest initiation and nest initiation quadratic term were kept as a baseline model in the second AIC model selection step.

| **Models** | ***k*** | **AICc** | **ΔAICc** | **ModelLik** | **Weight** | **LL** |
| --- | --- | --- | --- | --- | --- | --- |
| Nest.Init | 3 | 369.487 | 0.000 | 1.000 | 0.730 | -181.607 |
| Nest.Init^2^ | 4 | 371.481 | 1.994 | 0.369 | 0.270 | -181.510 |
| Null | 2 | 416.961 | 47.475 | 0.000 | 0.000 | -206.413 |
| Age | 3 | 417.117 | 47.630 | 0.000 | 0.000 | -205.422 |
| Nest.Year | 4 | 421.091 | 51.604 | 0.000 | 0.000 | -206.315 |

Table S7. Second AIC model selection models to identify pathogen variables affecting clutch size during the first nesting attempt of female wild turkeys captured and monitored from 2018–2020 in Maine. All models contain nest initiation and nest initiation quadratic term as explanatory variables, following Table S6 (for simplification, we only show the quadratic term). Variables determined to be significant upon interpretation of coefficients and their 95% confidence intervals in a model containing all supported variables (<2 ΔAICc) are italicized.

| **Model** | ***k*** | **AICc** | **ΔAICc** | **ModelLik** | **Weight** | **LL** |
| --- | --- | --- | --- | --- | --- | --- |
| Nest.Init^2^ + *LPDV* | 5 | 362.656 | 0.000 | 1.000 | 0.411 | -175.979 |
| Nest.Init^2^ + LPDV + Age | 6 | 363.169 | 0.512 | 0.774 | 0.318 | -175.090 |
| Nest.Init^2^ + LPDV + Age + LPDV*age | 7 | 365.404 | 2.748 | 0.253 | 0.104 | -175.036 |
| Nest.Init^2^ + Coinf | 7 | 365.976 | 3.320 | 0.190 | 0.078 | -175.321 |
| Nest.Init^2^ + Coinf + Age | 8 | 366.248 | 3.591 | 0.166 | 0.068 | -174.256 |
| Nest.Init^2^ + Coinf + Coinf*Age | 10 | 369.970 | 7.313 | 0.026 | 0.011 | -173.627 |
| Nest.Init^2^ | 4 | 371.481 | 8.824 | 0.012 | 0.005 | -181.510 |
| Nest.Init^2^ + Age | 5 | 373.169 | 10.513 | 0.005 | 0.002 | -181.236 |
| Nest.Init^2^ + REV | 5 | 373.176 | 10.519 | 0.005 | 0.002 | -181.239 |
| Nest.Init^2^ + REV + Age | 6 | 374.915 | 12.258 | 0.002 | 0.001 | -180.963 |
| Nest.Init^2^ + REV + Age + REV*Age | 7 | 376.991 | 14.334 | 0.001 | 0.000 | -180.829 |
| Null | 2 | 416.961 | 54.305 | 0.000 | 0.000 | -206.413 |

Table S8. Initial AIC model selection comparing univariate models to identify relevant non-pathogen variables affecting clutch size during the second nesting attempt of female wild turkeys captured and monitored from 2018–2020 in Maine. Age was kept as a baseline null model in the second AIC model selection step.

| **Model** | ***k*** | **AICc** | **ΔAICc** | **ModelLik** | **Weight** | **LL** |
| --- | --- | --- | --- | --- | --- | --- |
| Null | 2 | 61.755 | 0.000 | 1.000 | 0.566 | -28.332 |
| Age | 3 | 63.566 | 1.811 | 0.404 | 0.229 | -27.583 |
| Nest.Init | 3 | 64.386 | 2.631 | 0.268 | 0.152 | -27.993 |
| Nest.Init^2^ | 4 | 67.550 | 5.795 | 0.055 | 0.031 | -27.553 |
| Nest.Year | 4 | 68.312 | 6.557 | 0.038 | 0.021 | -27.934 |

Table S9. Second AIC model selection models to identify pathogen variables affecting clutch size during the second nesting attempt of female wild turkeys captured and monitored from 2018–2020 in Maine. All models contain turkey age as an explanatory variable, following Table S8. No variables were determined to be significant upon interpretation of coefficients and their 95% confidence intervals in a model containing all supported variables (<2 ΔAICc).

| **Model** | ***k*** | **AICc** | **ΔAICc** | **ModelLik** | **Weight** | **LL** |
| --- | --- | --- | --- | --- | --- | --- |
| Null | 2 | 61.755 | 0.000 | 1.000 | 0.533 | -28.332 |
| Age | 3 | 63.566 | 1.811 | 0.404 | 0.215 | -27.583 |
| Age + LPDV | 4 | 65.053 | 3.298 | 0.192 | 0.102 | -26.304 |
| Age + LPDV + LPDV*Age | 4 | 65.053 | 3.298 | 0.192 | 0.102 | -26.304 |
| Age + REV | 4 | 66.944 | 5.189 | 0.075 | 0.040 | -27.250 |
| Age + REV + REV*Age | 5 | 71.075 | 9.320 | 0.009 | 0.005 | -26.788 |
| Age + Coinf | 6 | 74.615 | 12.860 | 0.002 | 0.001 | -25.308 |
| Age + Coinf + Coinf*Age | 6 | 74.615 | 12.860 | 0.002 | 0.001 | -25.308 |

Table S10. Initial AIC model selection comparing univariate models to identify relevant non-pathogen variables affecting nest initiation during the first nesting attempt of female wild turkeys captured and monitored from 2018–2020 in Maine. Nest year was kept as a baseline null model in the second AIC model selection step.

| **Model** | ***k*** | **AICc** | **ΔAICc** | **ModelLik** | **Weight** | **LL** |
| --- | --- | --- | --- | --- | --- | --- |
| Nest.Year | 4 | 802.023 | 0.000 | 1.000 | 0.832 | -396.799 |
| Null | 2 | 806.160 | 4.137 | 0.126 | 0.105 | -401.017 |
| Age | 3 | 807.194 | 5.171 | 0.075 | 0.0627 | -400.470 |

Table S11. Second AIC model selection models to identify pathogen variables affecting nest initiation during the first nesting attempt of female wild turkeys captured and monitored from 2018–2020 in Maine. All models contain nest year as explanatory variable, following Table S10. Variables determined to be significant upon interpretation of coefficients and their 95% confidence intervals in a model containing all supported variables (<2 ΔAICc) are italicized.

| **Model** | **k** | **ΔAICc** | **Delta_AICc** | **ModelLik** | **Weight** | **LL** |
| --- | --- | --- | --- | --- | --- | --- |
| *Nest.Year* | 4 | 802.023 | 0.000 | 1.000 | 0.292 | -396.799 |
| Nest.Year + Age | 5 | 803.000 | 0.978 | 0.613 | 0.179 | -396.178 |
| Nest.Year + REV | 5 | 803.717 | 1.695 | 0.429 | 0.125 | -396.536 |
| Nest.Year + LPDV | 5 | 803.761 | 1.738 | 0.419 | 0.122 | -396.558 |
| Nest.Year + LPDV + Age | 6 | 804.436 | 2.413 | 0.299 | 0.087 | -395.761 |
| Nest.Year + REV + Age | 6 | 804.743 | 2.721 | 0.257 | 0.075 | -395.915 |
| Null | 2 | 806.160 | 4.137 | 0.126 | 0.037 | -401.017 |
| Nest.Year + LPDV + Age + LPDV*Age | 7 | 806.753 | 4.730 | 0.094 | 0.027 | -395.761 |
| Nest.Year + REV + Age + REV*Age | 7 | 807.042 | 5.020 | 0.081 | 0.024 | -395.906 |
| Nest.Year + Coinf | 7 | 807.604 | 5.581 | 0.061 | 0.018 | -396.187 |
| Nest.Year + Coinf + Age | 8 | 808.410 | 6.387 | 0.041 | 0.012 | -395.405 |
| Nest.Year + Coinf + Age + Coinf*Age | 10 | 813.209 | 11.186 | 0.004 | 0.001 | -395.354 |

Table S12. Initial AIC model selection comparing univariate models to identify relevant non-pathogen variables affecting nest initiation during the second nesting attempt of female wild turkeys captured and monitored from 2018–2020 in Maine. Age was kept as the baseline model in the second AIC model selection step.

| **Model** | ***k*** | **AICc** | **ΔAICc** | **ModelLik** | **Weight** | **LL** |
| --- | --- | --- | --- | --- | --- | --- |
| Age | 3 | 129.353 | 0.000 | 1.000 | 0.781 | -60.753 |
| Null | 2 | 132.161 | 2.808 | 0.246 | 0.192 | -63.652 |
| Nest.Year | 4 | 136.057 | 6.705 | 0.035 | 0.0273 | -62.362 |

Table S13. Second AIC model selection to identify pathogen variables affecting nest initiation during the second nesting attempt of female wild turkeys captured and monitored from 2018–2020 in Maine. All models contain age as an explanatory variable, following Table S12. Interaction between LPDV and age could not be assessed due to zero juveniles with LPDV and the 4-category coinfection variable and age could not be assessed due to small sample size resulting in category singularities. Variables determined to be significant upon interpretation of coefficients and their 95% confidence intervals in a model containing all supported variables (<2 ΔAICc) are italicized.

| **Model** | ***k*** | **AICc** | **ΔAICc** | **ModelLik** | **Weight** | **LL** |
| --- | --- | --- | --- | --- | --- | --- |
| *Age* | 3 | 129.353 | 0.000 | 1.000 | 0.585 | -60.753 |
| Null | 2 | 132.161 | 2.808 | 0.246 | 0.126 | -63.652 |
| Age + LPDV | 4 | 132.485 | 3.132 | 0.209 | 0.122 | -60.576 |
| Age + REV | 4 | 132.554 | 3.201 | 0.202 | 0.118 | -60.610 |
| Age + REV + REV*Age | 5 | 134.591 | 5.238 | 0.073 | 0.043 | -59.568 |
| Age + Coinf | 6 | 139.010 | 9.658 | 0.008 | 0.005 | -59.305 |
|  |  |  |  |  |  |  |

Table S14. Initial AIC model selection comparing univariate models to identify relevant non-pathogen variables affecting nesting propensity during the first nesting attempt of female wild turkeys captured and monitored from 2018–2020 in Maine. Nest year was kept as the baseline model in the second AIC model selection step.

| **Model** | ***k*** | **AICc** | **ΔAICc** | **ModelLik** | **Weight** | **LL** |
| --- | --- | --- | --- | --- | --- | --- |
| Nest.Year | 3 | 41.472 | 0.000 | 1.000 | 0.553 | -17.412 |
| Null | 1 | 42.575 | 1.103 | 0.576 | 0.318 | -20.236 |
| Age | 2 | 44.380 | 2.908 | 0.234 | 0.129 | -20.032 |

Table S15. Second AIC model selection to identify pathogen variables affecting nesting propensity during the first nesting attempt of female wild turkeys captured and monitored from 2018–2020 in Maine. All models contain nest year as an explanatory variable, following Table S14. Interaction between the 4-category coinfection variable and age could not be assessed due to small sample size resulting in category singularities. No variables were determined to be significant upon interpretation of coefficients and their 95% confidence intervals in a model. containing all supported variables (<2 ΔAICc).

| **Model** | **k** | **AICc** | **ΔAICc** | **ModelLik** | **Weight** | **LL** |
| --- | --- | --- | --- | --- | --- | --- |
| Nest.Year + REV | 4 | 40.983 | 0.000 | 1.000 | 0.356 | -15.936 |
| Null | 1 | 42.575 | 1.592 | 0.451 | 0.161 | -20.236 |
| Nest.Year + REV + Age | 5 | 43.512 | 2.529 | 0.282 | 0.101 | -15.899 |
| Nest.Year + Coinf | 5 | 43.546 | 2.564 | 0.278 | 0.099 | -15.916 |
| Nest.Year + LPDV | 4 | 43.649 | 2.667 | 0.264 | 0.094 | -17.269 |
| Nest.Year + Age | 4 | 43.931 | 2.948 | 0.229 | 0.082 | -17.410 |
| Nest.Year + LPDV + Age + LPDV*Age | 6 | 45.817 | 4.835 | 0.089 | 0.032 | -15.673 |
| Nest.Year + LPDV + Age | 5 | 46.247 | 5.265 | 0.072 | 0.026 | -17.266 |
| Nest.Year + Coinf + Age | 6 | 46.252 | 5.270 | 0.072 | 0.026 | -15.891 |
| Nest.Year + REV + Age + REV*Age | 6 | 46.268 | 5.285 | 0.071 | 0.025 | -15.899 |
|  |  |  |  |  |  |  |
|  |  |  |  |  |  |  |
|  |  |  |  |  |  |  |
|  |  |  |  |  |  |  |
|  |  |  |  |  |  |  |

Table S16. Initial AIC model selection comparing univariate models to identify relevant non-pathogen variables affecting nesting propensity during the second nesting attempt of female wild turkeys captured and monitored from 2018–2020 in Maine. The null model was kept as the baseline model in the second AIC model selection step.

| **Model** | ***k*** | **AICc** | **ΔAICc** | **ModelLik** | **Weight** | **LL** |
| --- | --- | --- | --- | --- | --- | --- |
| Null | 1 | 2.190 | 0.000 | 1.000 | 0.725 | 0.000 |
| Age | 2 | 4.600 | 2.410 | 0.300 | 0.217 | 0.000 |
| Nest.Year | 3 | 7.263 | 5.073 | 0.079 | 0.057 | 0.000 |

Table S17. Second AIC model selection to identify pathogen variables affecting nesting propensity during the second nesting attempt of female wild turkeys captured and monitored from 2018–2020 in Maine. No variables were retained from the first model (Table S16). Due to small sample size, we only assessed univariate pathogen variables. Coincidentally, infection LPDV and REV mirrored each other where 18 were infected with LPDV and 5 were not, and 5 were infected with REV and 18 were not. No individual in this analysis was infected with REV alone (hence “Coinf” having 3 parameters). The null model performed the best, likely due to low sample size.

| **Model** | ***k*** | **AICc** | **ΔAICc** | **ModelLik** | **Weight** | **LL** |
| --- | --- | --- | --- | --- | --- | --- |
| Null | 1 | 2.190 | 0.000 | 1.000 | 0.596 | 0.000 |
| LPDV | 2 | 4.600 | 2.410 | 0.300 | 0.179 | 0.000 |
| REV | 2 | 4.600 | 2.410 | 0.300 | 0.179 | 0.000 |
| Coinf | 3 | 7.263 | 5.073 | 0.079 | 0.047 | 0.000 |
|  |  |  |  |  |  |  |

Table S18. Initial AIC model selection comparing univariate models to identify relevant non-pathogen variables affecting egg hatch rate of successful nests of female wild turkeys captured and monitored from 2018–2020 in Maine. Nest initiation and nest initiation quadratic term were kept as a baseline model in the second AIC model selection step.

| **Model** | ***k*** | **AICc** | **ΔAICc** | **ModelLik** | **Weight** | **LL** |
| --- | --- | --- | --- | --- | --- | --- |
| Nest.Init | 3 | -38.144 | 0.000 | 1.000 | 0.391 | 22.447 |
| Null | 2 | -36.992 | 1.152 | 0.562 | 0.219 | 20.678 |
| Nest.Init^2^ | 4 | -36.296 | 1.848 | 0.397 | 0.155 | 22.793 |
| Age | 3 | -35.402 | 2.742 | 0.254 | 0.099 | 21.076 |
| Nest.Year | 4 | -34.926 | 3.218 | 0.200 | 0.078 | 22.108 |
| Attempt | 4 | -34.319 | 3.825 | 0.148 | 0.058 | 21.805 |
|  |  |  |  |  |  |  |

Table S19. Second AIC model selection to identify pathogen variables affecting hatch rate of successful nests of female wild turkeys captured and monitored from 2018–2020 in Maine. All models contain nest initiation and nest initiation quadratic term as explanatory variables, following Table S18 (for simplification, we only show the quadratic term). Due to small sample size, we only assessed univariate pathogen variables as an additive variable with nest initiation. No variables were determined to be significant upon interpretation of coefficients and their 95% confidence intervals in a model containing all supported variables (<2 ΔAICc).

| **Model** | ***k*** | **AICc** | **ΔAICc** | **ModelLik** | **Weight** | **LL** |
| --- | --- | --- | --- | --- | --- | --- |
| Null | 2 | -36.992 | 0.000 | 1.000 | 0.404 | 20.678 |
| Nest.Init^2^ | 4 | -36.296 | 0.696 | 0.706 | 0.285 | 22.793 |
| Nest.Init^2^ + LPDV | 5 | -34.888 | 2.103 | 0.349 | 0.141 | 23.444 |
| Nest.Init^2^ + REV | 5 | -34.395 | 2.596 | 0.273 | 0.110 | 23.198 |
| Nest.Init^2^ Coinf | 7 | -33.138 | 3.854 | 0.146 | 0.059 | 25.569 |
